# Metabolomics-based response of *Salmonella* to desiccation stress and skimmed milk powder storage

**DOI:** 10.3389/fmicb.2023.1092435

**Published:** 2023-02-23

**Authors:** Shaoting Li, Yingqi Chen, Ji Zeng, Haiyan Zeng, Zhuolin Ma, Siyi Chen, Yuheng Yang, Hongmei Zhang

**Affiliations:** College of Biological and Pharmaceutical Science, Guangdong University of Technology, Guangzhou, China

**Keywords:** *Salmonella*, metabolomics, desiccation, LMFs, stress responses

## Abstract

The strong survival ability of *Salmonella* in low-moisture foods (LMFs) has been of public concern, and is considered a threat to people’s health. Recently, the development of omics technology has promoted research on the molecular mechanisms of the desiccation stress response of pathogenic bacteria. However, multiple analytical aspects related to their physiological characteristics remain unclear. We explored the physiological metabolism changes of *S. enterica* Enteritidis exposed to a 24 h-desiccation treatment and a subsequent 3-month desiccation storage in skimmed milk powder (SMP) with an approach of gas chromatography–mass spectrometry (GC–MS) and ultra-performance liquid chromatography-Q Exactive-mass spectrometry (UPLC-QE-MS). A total of 8,292 peaks were extracted, of which 381 were detected by GC–MS and 7,911 peaks were identified by LC–MS/MS, respectively. Through analyses of differentially expressed metabolites (DEMs) and key pathways, a total of 58 DEMs emerged from the 24 h-desiccation treatment, which exhibited the highest relevance for five metabolic pathways, involving glycine, serine, and threonine metabolism, pyrimidine metabolism, purine metabolism, vitamin B6 metabolism, and pentose phosphate pathway. After 3-month SMP storage, 120 DEMs were identified, which were related to several regulatory pathways including arginine and proline metabolism, serine and threonine metabolism, β-alanine metabolism, glycerolipid metabolism, and glycolysis. The analyses of key enzyme activities of XOD, PK, and G6PDH and ATP content provided further evidence that supported the metabolic responses such as nucleic acid degradation, glycolysis, and ATP production played an important role in *Salmonella*’s adaptation to desiccation stress. This study enables a better understanding of metabolomics-based responses of *Salmonella* at the initial stage of desiccation stress and the following long-term adaptive stage. Meanwhile, the identified discriminative metabolic pathways may serve as potentially useful targets in developing strategies for the control and prevention of desiccation-adapted *Salmonella* in LMFs.

## 1. Introduction

Since ancient times, high-moisture foods are processed through traditional preservation methods, i.e., by adding sugar or salt to create low water activity (*a_w_*) conditions for microorganisms and prolong the food expiration date (Beuchat et al., 2013). Generally, those foods with an a_w_ below 0.85 are referred to as low-moisture foods (LMFs), such as milk powder, chocolate, nuts, pistachios, etc. (Young et al., 2015). It is generally acknowledged that a low a_w_ environment can inhibit the growth of pathogenic microorganisms. However, LMFs are frequently implicated in outbreaks of salmonellosis. Foodborne *Salmonella* outbreaks have been associated with a variety of LMFs including skimmed milk powder (Julseth et al., 1969), powdered infant formula (Jourdan-da Silva et al., 2018; Jiang et al., 2020), pistachios (Farakos et al., 2018; Haendiges et al., 2021), peanut butter, flour, dried vegetables, etc. (Sánchez-Maldonado et al., 2018). *Salmonella enterica* serotype Enteritidis (*S. Enteritidis*) is one of the most frequently reported serotypes that have been associated with foodborne salmonellosis outbreaks in China. Acquired tolerance to multiple stressors in desiccated *S. Enteritidis* have been previously reported (Gruzdev et al., 2011). The survival and persistence of *Salmonella* in LMFs may attribute to its strong tolerance to unfavorable conditions, which has become a topic of high relevance in the field of public health.

Various factors have a decisive impact on the survival of *Salmonella* in LMFs. The complex processes during the LMFs production subject *Salmonella* to a diversity of environmental stress, such as desiccation, heat treatment, pH regulation, and hydrogen peroxide stimulation (Andino and Hanning, 2015). During these process conditions, *Salmonella* is able to induce cross-resistance and prolong its survival in the food production chain (Gruzdev et al., 2011). Previous research has emphasized that storage conditions of LMFs, including storage time (Blessington et al., 2013; Sekhon et al., 2021), ambient temperature (Sirsat et al., 2011), and humidity (Keller et al., 2013), also have a critical influence on the long-term survival of *Salmonella*. Moreover, divergent responses of *Salmonella* to desiccation storage have been shown in foods with different nutritional or component content. For instance, fat (Podolak et al., 2010) and sugar (Olaimat et al., 2020) are both extensively perceived as protectants for microorganisms. It is widely regarded that the current inactivation methods for foodborne pathogens in LMFs are still limited, with concurrent *Salmonella* infections of generally long duration and high severity, which is a tremendous threat to people’s health.

In response to dehydration stress, *Salmonella* has developed highly sophisticated mechanisms, involving the accumulation of osmotic protectants (Wood et al., 2001), regulation of stress proteins and membrane porins (Graeme-Cook et al., 1989; McMeechan et al., 2007), change of cell membrane composition (Burgess et al., 2016), the transformation of the VBNC state (Lesn et al., 2000), and so on. Presently, emerging omics technology is widely utilized in the food safety field. This allows researchers to better understand the stress response of pathogens. In previous studies associated with LMFs, RNA-sequencing was applied to detect desiccation-induced genes of *Salmonella* (Gruzdev et al., 2012; Mandal and Kwon, 2017). In Deng’s research, the transcripts of *Salmonella* in peanut oil were identified through transcriptome sequencing (Deng et al., 2012). Besides, Crucello et al. revealed the genetic and adaptive differences of *Salmonella* Typhimurium in four LMFs through transcriptome analysis (Crucello et al., 2019). Transcriptomics can reveal complex biological pathways for *Salmonella* desiccation adaptation by comprehensively studying gene expression and regulation. In these reports, there exist discrepancies possibly due to the differences in multiple factors, such as the variable serotypes of strains, the experimental desiccation settings, and genomic techniques. In the last decades, metabolomics becomes an effective tool for comprehensive profiling of metabolites. Metabolomics involves quantitative analysis of the overall changes of small-molecule metabolites under certain conditions, which can reveal whether metabolites are linked to physiological changes. Thus, metabolomics can aid in the analysis and identification of metabolite profiles and the explanation of response mechanisms of *Salmonella*’s adaption under low water activity stress. Although metabolomic analysis has been performed to investigate the antimicrobial mechanism of *Salmonella* on foods treated with organic acids (Guo et al., 2022), metabolomics-based mechanisms describing the adaptability of *Salmonella* during desiccation storage have been rarely reported.

Aiming to fill the information gaps in this field, the present study explored the physiological and metabolic changes of *S. Enteritidis* desiccated and stored in the skimmed milk powder (SMP) by approaches of GC–MS and LC–MS/MS. In this study, the desiccation-induced responses and adaptive mechanisms of *S. Enteritidis* in LMFs were further elucidated, which would provide useful hints for the development of targeted measures for the control and prevention of desiccation-adapted *Salmonella* in the production and long-term storage of LMFs.

## 2. Materials and methods

### 2.1. Strains and growth condition

The *S. Enteritidis* strain CICC 21482, purchased from the China Center of Industrial Culture Collection (Beijing, China), was used for the study. The stock culture stored at −80°C in the refrigerator was initially resuscitated in fresh Trypticase Soy Broth supplemented with 0.6% (w/v) yeast extract (TSBYE, Huankai Biotechnology Co., Guangzhou, China). After incubating at 37°C for 24 h, 100 μL suspensions were transferred to a Trypticase Soy Agar Plate with 0.6% yeast extract (TSAYE, Huankai Biotechnology Co., Guangzhou, China) for overnight culture in the incubator at 37°C. Bacteria cells were recovered by adding 1 mL sodium phosphate buffer (0.5 M, pH 7.0) to the agar surface and gently suspending the cells using a sterile plate spreader (Keller et al., 2013). The obtained bacterial suspension had a final cell density of approximately 10^10^ CFU/mL.

### 2.2. Desiccation and milk powder storage

The prepared bacterial suspensions of *S. Enteritidis* were referred to as the 0-samples. Dehydration and SMP storage of *Salmonella* were performed as previously described in the literature (Chen et al., 2021). First, the silica gels were initially dehydrated in a 108°C oven to ensure complete water removal and irradiated with UV light for 30 min to kill the background bacteria. For the dehydration process, the bacterial suspension was dispensed drop-wise on the 0.22-μm-pore-size nitrocellulose membrane filter disks (25 mm; Millipore, Sigma, Burlington, MA), where each drop contained 100 μL. Then, the membranes were laid on several 15-cm Petri dishes without cover, followed by a 24 h-desiccation treatment in drying jars containing anhydrous silica gels, to reduce the a_w_ of the bacteria to approximately 0.3. Water activity was measured with an intelligent water activity meter (HD-4, Huake Instrument and Meter CO., Ltd., Wuxi, China). Bacterial cells collected after 24 h desiccation were referred to as the 24 h samples.

SMP was inoculated with *S. Enteritidis* for investigation of their long-term survival. SMP (Anchor dairy, Auckland, New Zealand) was purchased from a local market in Guangzhou, China. Before the storage experiments, the ISO 6579 method was used to confirm the absence of *Salmonella* spp. in SMP. Then, a total of 3 g SMP each were transferred into sterile bottles, in which the 24 h samples loaded on the nitrocellulose membrane were buried. One piece of the membrane was put in each bottle, in triplicate analysis, and subsequently stored in a drying jar containing anhydrous silica gels at 25°C for 3 months. The water activity was kept at 0.3 during the storage period. Bacterial cells collected after 3-month storage in SMP were referred to as the 3-month samples.

The membranes of the 24 h samples and the 3-month samples were, respectively, transferred to 10 mL sodium phosphate buffer (0.5 M, pH 7.0) for resuspension by vortexing for 5 min, to maximize the separation of bacterial cells and to minimize cell lysis. Then the bacterial cells were further diluted in buffered protein water, and plated on xylose lysine deoxycholate agar (XLD; Qingdao Haibo Biotechnology Co., Qingdao, China). Colonies were counted after 24 h incubation at 37°C. The samples were further adjusted to approximately 10^7^ CFU/mL in sodium phosphate buffer for further study.

### 2.3. Metabolite state being stabilized

To make sure the extracted metabolites truly reflected the physiological response to the specific treatment, the bacterial solution obtained at each experimental step was quenched (Cai et al., 2015). The volume of the culture solution containing 10^7^ cells was determined in the previous experiment. At each treatment, the cells on the membrane were resuspended in sodium phosphate buffer (0.5 M, pH 7.0), and the required solution volume was transferred into an Eppendorf tube. After centrifugation at 4°C, 1,000 × g for 10 min, the supernatant was removed, and the cell precipitate was rapidly quenched in liquid nitrogen for 30 s. Then, the samples were thawed slowly on ice and washed with 4°C sterile phosphate buffer saline (PBS) to remove the residual medium. All samples were stored at −80°C, with three biological replicates in separate batches.

### 2.4. Metabolite extraction and derivatization for GC-TOF-MS

The metabolomics analysis was performed based on a combined platform of GC–MS and LC–MS/MS to obtain a comprehensive metabolic profiling of *S. Enteritidis*. For GC–MS, different volumes of water were added to each sample to obtain a final bacterial concentration of 4 × 10^8^ CFU/mL. After vortexing, 200 μL of the homogenate was transferred to a 2 mL Eppendorf tube, respectively. Afterward, 800 μL of methanol/acetonitrile (1:1, v/v), containing ribitol (Sigma-Aldrich, St. Louis, Missouri) as the internal standard (IS) was supplemented, followed by grinding in a 35-Hz grinder (JXFSTPRP-24, Jingxin Industrial Development Co., Ltd., Shanghai, China) for 4 min and sonication in ice-water for 5 min. After three repetitions, the samples were kept at −40°C for an hour and then centrifuged at 12,000 rpm at 4°C for 15 min. The supernatant was carefully removed from the tube and dried in a vacuum concentrator.

The extracts were derivatized before GC–MS analysis. The extracts were blended with 30 μL of methoxyamine hydrochloride (Tokyo Chemical Industry Co. Ltd., Japan), diluted in pyridine to 20 mg/mL, and maintained at 80°C. After 30 min incubation, 40 μL Bis (trimethylsilyl) trifluoro acetamide (BSTFA) with 1% Trimethylchlorosilane (TMCS)(v/v) (Regis Technologies, Inc., Morton Grove, United States) was added to each sample. Then, the mixture was sequentially incubated at 70°C for 1.5 h for later analysis.

### 2.5. Detection method set-up for GC-TOF-MS

Samples were analyzed on an Agilent 7890B gas chromatography-time of flight mass spectrometer (7890B, Agilent Technologies, Inc., Wilmington, United States), equipped with an Agilent DB-5MS capillary column (30 m × 250 μm × 0.25 μm, Agilent Technologies, Inc., Wilmington, USA). Using helium as the carrier gas, 1 μL of each sample was injected in splitless mode, with the column flow rate of 1 mL/min and front inlet septum purge flow of 3 mL/min. The injector, transfer line, and ion source temperatures were 280, 280, and 250°C, respectively. The oven’s original temperature was 50°C and kept for 1 min, followed by a temperature ramped rate of 10°C/min to 310°C and then kept constant for 8 min. The electron energy was set at −70 eV. The mass spectrometry data were acquired in the full-scan mode over the m/z range of 50–500, at an acquisition rate of 12.5 spectra/s, and a solvent delay of 6.25 min. In the detection process, the first and the last samples of each sequence were the same samples to monitor system deviations based on the stability of the IS and the TIC of repeatedly injected samples.

For data processing, the Chroma TOF software (V4.3x, LECO Corporation, St. Joseph, MI, United States) was applied to analyze the raw mass spectral data, such as peak extraction, baseline adjustment, deconvolution, integration, and alignment (Kind et al., 2009). In the qualitative analyses of substances, mass spectrum and retention index matching were performed using the LECO-Fiehn Rtx5 database.

### 2.6. Metabolomics analysis by UHPLC-QE-MS

The remaining supernatant as described in “Metabolite extraction and derivatization for GC-TOF-MS” was transferred to an alternative tube that was used for LC–MS/MS detection. In this part, analysis was performed by an ultra-high performance liquid chromatography (UHPLC) system (Vanquish, Thermo Fisher Scientific Inc., New York, United States), equipped with a UPLC BEH Amide liquid chromatographic column (2.1 mm × 100 mm, 1.7 μm) coupled to a Q Exactive HFX mass spectrometer (Orbitrap MS, Thermo). Three micro liter of metabolite solutions was injected and eluted with the mobile phase consisting of 25 mmol/l each of ammonium acetate and ammonia hydroxide in water (pH = 9.75) (A) and acetonitrile (B), respectively.

The QE HFX mass spectrometer was used for its ability to acquire MS/MS spectra in information-dependent acquisition (IDA) mode controlled by the acquisition software (Xcalibur, Thermo Fisher Scientific Inc., New York, United States). In this mode, the acquisition software continuously evaluates the full-scan MS spectrum. The ESI source conditions were set as follows: sheath gas flow rate set to 25 Arb, auxiliary gas flow rate to 20 Arb, capillary temperature 350°C, full ms resolution to 60,000, MS/MS resolution to 7,500, collision energy to 10/30/60 in NCE mode, and spray Voltage to 3.6 kV (positive) or − 3.2 kV (negative), respectively (Wang et al., 2016).

For data processing, the raw data were converted into an mzXML format by the ProteoWizard software; the R package with the core of XCMS^1^ was utilized for data processing, i.e., peak identification, extraction, alignment, and integration (Smith et al., 2006). Substances were annotated by matching with the built-in BiotreeDB (V2.1) secondary mass spectrometry database, with the cutoff value of the algorithm score set to 0.3.

### 2.7. Metabolomics data pretreatment and statistical analysis

The original data generated by GC–MS and LC–MS/MS analysis required further noise removal, normalization, and standardization, which were conducted according to a previously published protocol (Dunn et al., 2011). After data processing, the Student’s *t*-test and multivariate analysis (MVA), including principal component analysis (PCA) and orthogonal partial least squares discriminant analysis (OPLS-DA), were performed using SIMCA software (V16.0.2, Sartorius Stedim Data Analytics AB, Umea, Sweden).

PCA was conducted after LOG conversion and centralization (CTR) data formatting along with modeling analysis, which contributed to visualizing metabolite differences. Similarly, OPLS-DA was carried out after LOG conversion and UV formatting; the objective of this modeling was to determine the first principal component from PCA. The quality of the OPLS-DA model was tested by a 7-fold cross-validation. Briefly, the values of *R*^2^*Y* (the interpretability of the model to the classification variable Y) and *Q*^2^ (the predictability of the model) obtained from cross-validation were used to evaluate the reliability and stability of the model. Model overfitting was ultimately assessed through the permutation test. This test indicates whether the arrangement order of the classification variables *Y* was changed randomly, and the corresponding OPLS-DA models were established 200 times to obtain the *R*^2^ and *Q*^2^ values of the random model.

### 2.8. Screening of differentially expressed metabolites

The critical value, obtained from the variable importance in the projection (VIP) of the established OPLS-DA model, summarized the contribution of each variable to the model (Li X. et al., 2018). Differential metabolites were screened according to the VIP value and the statistical value of significance (*P* value). In this work, two analysis groups were specified for the comparison, the desiccation group (24 h samples vs. 0-samples) and the storage group (3-month samples vs. 24 h samples), in which those metabolites with VIP > 1 and *p* < 0.05 (Student’s *t*-test) were considered as differentially expressed metabolites (DEMs) (Höcker et al., 2021). After preliminary screening, DEMs were classified based on HMBD^2^ and KEGG.^3^ To further explore the stress-induced metabolism difference and regulatory mechanisms of *Salmonella*, hierarchical cluster analysis, KEGG pathway annotation (Kanehisa and Goto, 2000), and metabolic pathway analysis of DEMs were carried out. Hierarchical cluster analysis was performed with Euclidean distances using R software.^4^ MetaboAnalyst software 3.0 was used to identify the top altered metabolic pathways (Xia et al., 2015).

### 2.9. Measurement of key enzyme activities and ATP content

Based on the metabolomics results, three key enzymes involved in essential pathways, including xanthine oxidase (XOD), pyruvate kinase (PK), and glucose 6-phosphate dehydrogenase (G6PDH), were examined for changes of their activities under desiccation stress. In brief, *Salmonella* cell suspensions at different sampling points were diluted with sodium phosphate buffer (0.5 M, pH 7.0) to obtain a final concentration of 10^7^ CFU/mL. The extraction solution was added according to the manufacturer’s instructions (Comin Biotechnology Co. Ltd., Suzhou, China). The mixture was ultrasonically broken with an ultrasonic ice bath (200 W, ultrasonic time 3 s at intervals of 10 s, and 30 times of ultrasound treatment). The supernatant was obtained by centrifugation at 8,000 × g and 4°C for 10 min and then incubated at room temperature for 30 min after adding the reaction reagent. UV spectrophotometric (UV–VIS, Thermo Fisher Scientific, United States) analysis was used to measure the concentration change of the reaction products catalyzed by specific enzymes. The characteristic absorption wavelength was 290 nm for XOD, and 340 nm for PK and G6PDH, respectively. The enzyme activity was determined in units of nmol/min/10^4^ cells.

In addition, the effect of continuous stress on the intracellular ATP content was also determined to further verify the regulation of metabolic pathways. *Salmonella* cell suspensions at different sampling points were centrifuged at 5,000 rpm for 5 min at 4°C, and then the supernatant was removed. Five milliliter of phosphate buffer saline solution (PBS, pH7.2) were added for resuspension. Cell suspensions were broken with ultrasound in an ice bath for 5 min. The supernatants were collected after centrifugation at 12,000 rpm for 20 min at 4°C. The ATP levels were assayed using an ATP assay kit (Jiancheng Bioengineering Institute, Nanjing, China). The results were analyzed by UV–VIS with an absorption wavelength of 636 nm. All assays were performed in biological triplicates.

The results were expressed as mean ± standard deviation. ANOVA was performed in SPSS 22.0 to determine significant differences between groups (*p* < 0.05).

## 3. Results and discussion

### 3.1. *Salmonella* survival under desiccation stress and SMP storage

Survival experiments of *Salmonella* under desiccation stress and after storage in SMP were conducted, in which the amount of dropped/collected bacterial suspensions was showed as colony-forming units (CFU) on the XLD plates. Representative colonies from XLD (black colonies) were confirmed to be *Salmonella* by PCR using primers that target a 94-bp segment of the *Salmonella*-specific *ttr* gene (GenBank accession no. AF 282268) (Malorny et al., 2004). The results showed that the number of bacteria loaded on the membrane before desiccation treatment (100 μL) was (4.26 ± 1.24) × 10^9^ CFU. After desiccation for 24 h, the strain population decreased to (2.07 ± 0.69) × 10^9^ CFU without significant difference (*p* > 0.05). *Salmonella* population in the 3-month samples was (6.55 ± 1.05) × 10^8^ CFU, which was slightly lower than that in the 24 h samples (*p* < 0.05). In addition, it was still absent of *Salmonella* in the non-inoculated control group. It is obvious that *Salmonella* developed strong tolerance to desiccation stress and had high survival rate during SMP storage.

In food industry, desiccation and hyperosmotic treatments for effective inhibition of the growth of microorganisms are the most common methods of food preservation. However, it has been widely reported that *Salmonella* can survive desiccation for long periods under low water activity conditions (Dhaliwal et al., 2021; Morasi et al., 2022). Consistent with the previous work, *Salmonella* in this study remained stable population during the dehydration treatment and SMP storage, which indicated that *Salmonella* had developed adaptability to low moisture conditions. Notably, in this study, bacteria were inoculated while in transition from broth to the solid medium. Cuny et al. indicated that the transition of cells from liquid to solid medium would induce the expression of several stress regulators (Cuny et al., 2007), which enhance their ability to resist environmental stress.

### 3.2. Stability of the GC–MS and LC–MS/MS system

From the GC–MS and LC–MS/MS analyses, a total of 8,292 peaks were extracted, of which 381 were detected by GC–MS and 7,911 peaks were identified by LC–MS/MS. After the raw data processing, 8,277 peaks were retained. To ensure the reliability of the data, quality control (QC) was conducted during the analysis to monitor instrument stability, internal standard (IS) response, and substance residue. In this experiment, the second tube of the 24 h sample was used as the repeated injection sample. As shown in the total ion chromatogram (TIC) in Supplementary Figure 1A, the peak retention time and peak area of the repeated sample basically overlapped, indicating that the instrument was stable. The peak area RSD of the IS in the repeated sample was 1.41% (data not shown), which confirmed that the data acquisition of the system was stable. Blank samples allowed to detection of residual substances in the test process. It can be seen from Supplementary Figure 1B that there was no significant peak detected in the blank sample, illustrating that substance residues were negligible and there was no cross-contamination between samples. Regarding LC–MS/MS, the overlayed TICs of both negative (NEG) mode and positive (POS) mode for all samples, analyzed separately and presented in Supplementary Figures 2, 3, indicate the stability of the system.

### 3.3. Metabolomic chemometric analysis based on GC–MS and LC–MS/MS

**Figure 1:**
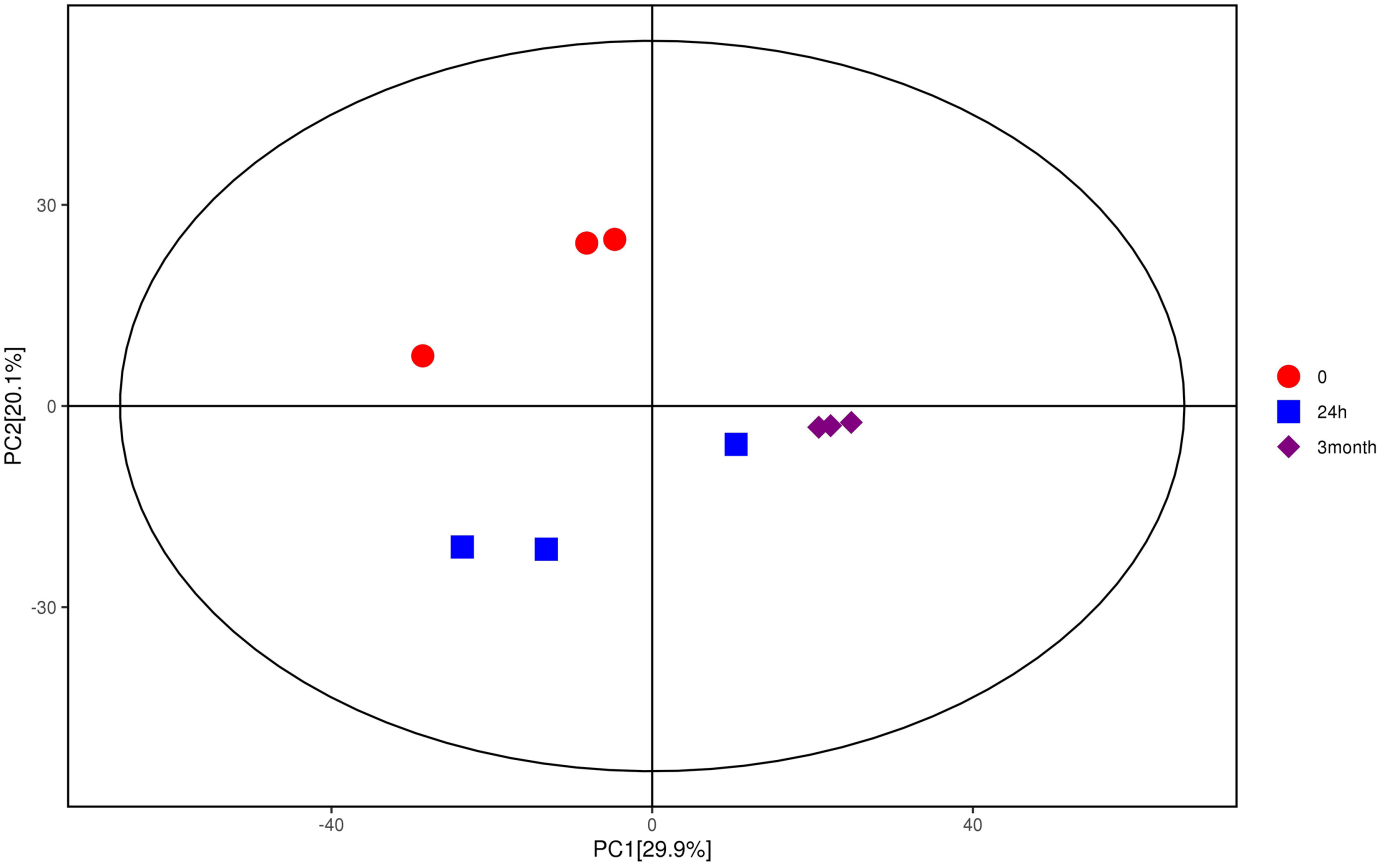
Score scatter plot for PCA model of metabolites of *Salmonella* after desiccation treatment and SMP storage. The *x*-axis PC1 and *y*-axis PC2 represents the aggregation of the first and second principal components, respectively. The color and shape of the scatters represent the experimental grouping of the samples. The samples are all within the 95% confidence interval (Hotelling’s *t*-squared ellipse).

Multivariate analysis, including PCA and OPLS-DA, was performed based on the metabolite data obtained by GC–MS and LC–MS/MS. As shown in Figure 1, samples with different groups showed distinctive separations, demonstrating the differences in metabolite profile of *Salmonella* induced by desiccation treatment and long-term storage in SMP. In the current model, the first ranked principal component (PC1) accounted for 29.9%, broadly separated the 0-samples and the 24 h samples from the 3-month samples, while the interpretability of the second principal component (PC2) to sample variance was 20.1%. The OPLS-DA score plot (Supplementary Figures 4A,B) also exhibited an obvious separations in the sample treatment as shown by the distinctly different abscissa values between control and treatment samples. The permutation test (Supplementary Figures 4C,D) showed that the *Q*^2^-value of the random model was all less than that of the original model, and the slope of the regression line of *Q*^2^ is positive, indicating that the original model is robust without overfitting.

### 3.4. Identification of significant differentially expressed metabolites

**Figure 2:**
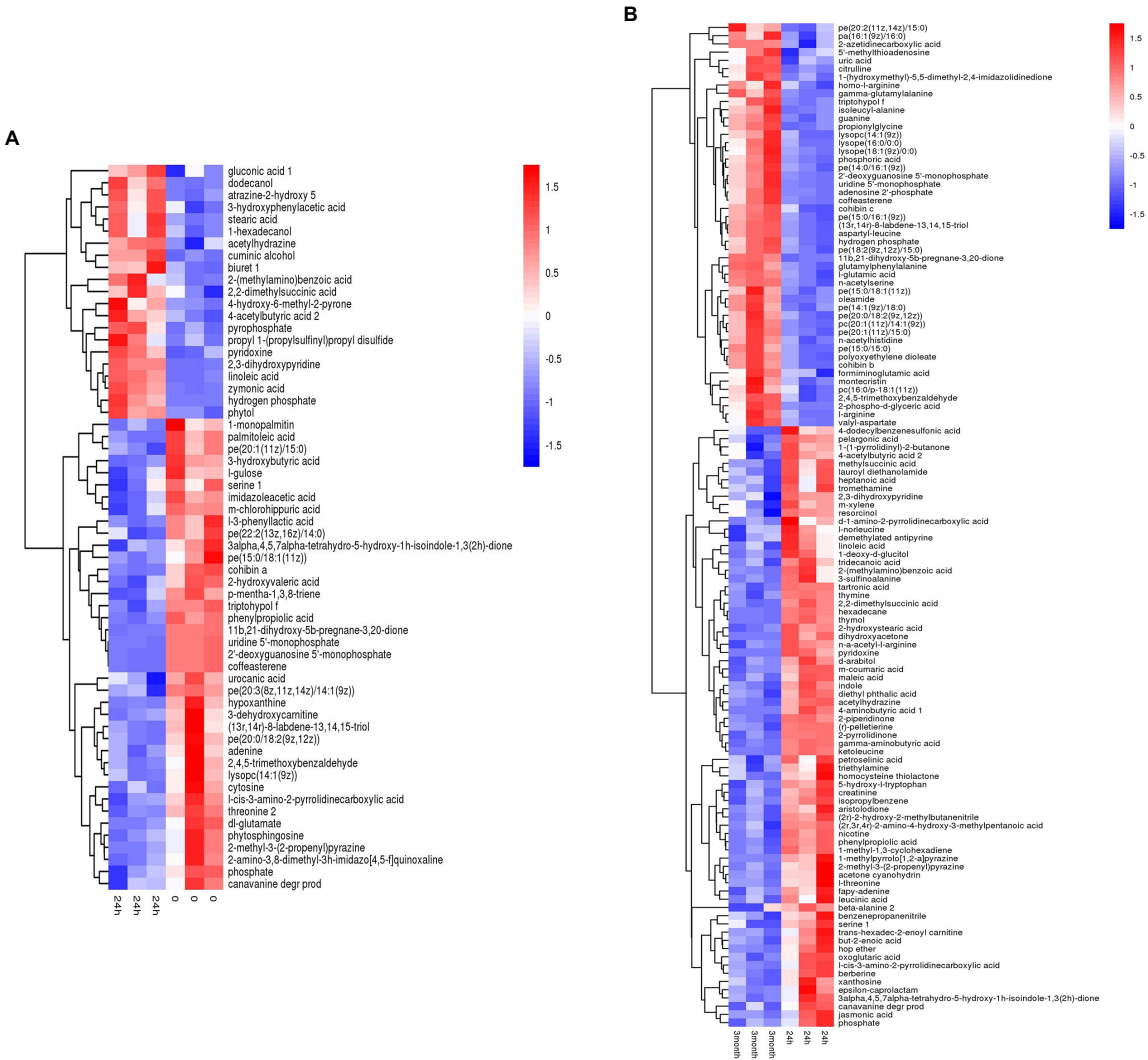
Heatmap of hierarchical clustering analysis of differentially expressed metabolites induced by **(A)** desiccation treatment and **(B)** SMP storage.

Based on the metabolites screening parameters (*p* < 0.05 and VIP > 1), 58 and 120 DEMs were identified in the desiccation group and the storage group, respectively, as listed in Supplementary Tables 1, 2. It should be emphasized that the DEMs were determined by pairwise comparison either between the 24 h samples and 0-samples, or between the 3-month samples and 24 h samples, which assured that the obtained DEMs came from the process of desiccation treatment or SMP storage, respectively. Moreover, compared with the amount of *Salmonella* in SMP (~10^9^ CFU/g), the number of the background bacteria in SMP is almost negligible (<10^2^ CFU/g, date not shown), thus the difference in metabolite profile could be attributed to *S. Enteritidis* inoculated in SMP, rather than the background bacteria. The screened metabolites were visualized in the form of volcano plots (Supplementary Figure 5). Hierarchical cluster analysis was applied to gather the DEMs with similar expression patterns in the regulatory pathway, and distinguish those with distinctive functions, which was helpful to extract the variation characteristics of metabolites between samples. The heatmap results presented in Figure 2 show a clear distinction between up-regulated and down-regulated metabolites.

Desiccation-altered metabolites were categorized into multiple types of materials, containing amino acids, nucleic acids, carbohydrates, organic acids, inorganic salts, alcohols, fatty acids, lipids, and others. Among them, amino acids, nucleic acids, lipids, and a large portion of organic acids were down-regulated, while inorganic salts, fatty acids, and alcohols were up-regulated. However, during the storage in SMP, *Salmonella* activated long-term adaptive responses by regulating more kinds of metabolites, such as amino acids, nucleic acids, fatty acids, carbohydrates, inorganic salts, organic acids, peptides, lipids, and others. This illustrated that complicated regulation mechanisms of *Salmonella* were present during its adaptation in LMFs (Morasi et al., 2022). A detailed profiling of all DEMs obtained from the aforementioned between-group comparisons for all three groups (i.e., the 0-, 24 h, and 3-month samples) was shown in Supplementary Figure 6, which exhibited clear changes of metabolite pattern in either the desiccation group or the storage group when compared with the 0-samples. The difference in the metabolite levels indicated considerable metabolic alterations had occurred in *Salmonella* from the initial stage of desiccation stress to the long-term adaptive stage.

### 3.5. KEGG annotation and metabolic pathway analysis

The identified DEMs were mapped to the metabolic pathway network based on the KEGG database. The DEMs induced by desiccation treatment were annotated to 12 predicted pathways (Supplementary Table 1), while the DEMs induced by SMP storage were annotated to 20 predicted pathways (Supplementary Table 2). As listed in Supplementary Table 1, the desiccation-related pathways primarily belong to amino acid-, nucleotide-, and energy metabolism, fatty acid synthesis, ABC transporter, cofactor biosynthesis, etc. In comparison with the desiccation group, more metabolic pathways were mapped in the group of SMP storage (Supplementary Table 2), which involve amino acid biosynthesis, glyceride metabolism, carbohydrate metabolism, population sensitivity, etc.

**Figure 3:**
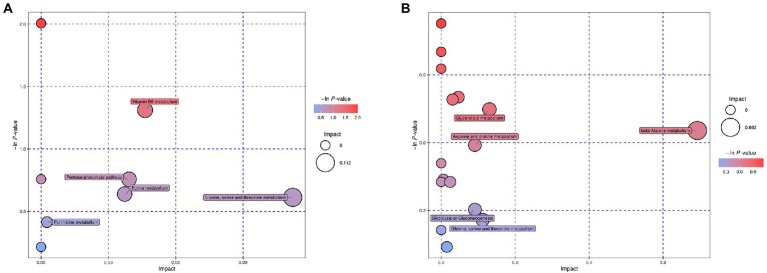
Bubble plots of pathway analysis of *Salmonella* after **(A)** desiccation treatment and **(B)** SMP storage. The circle size corresponds to impact values (*x*-axis) and the color reflects -ln *p*-values (*y*-axis). The pathway impact values were calculated from pathway topology analysis, which uses betweenness centrality measures to estimate node importance.

Enrichment analysis of the mapped metabolic pathways were further conducted to explore critical pathways with the highest correlation to metabolite differences (Figure 3). Metabolic pathway analysis of the desiccation group led to significant enrichment of glycine, serine, and threonine metabolism, vitamin B6 metabolism, purine metabolism, pyrimidine metabolism, and pentose phosphate pathway (Figure 3A). In contrast, the group of SMP storage showed different regulatory pathways (Figure 3B), which may be closely related to its adaptability in low water activity conditions, including glycerolipid metabolism β-alanine metabolism, arginine and proline metabolism, glycine, serine, and threonine metabolism, and glycolysis/gluconeogenesis.

### 3.6. Metabolic responses of *Salmonella enteric* to desiccation stress

Studies have revealed that when cells are exposed to environmental stress or nutrient deficiency, substances produced in the amino acid catabolism can be used as alternative carbon/nitrogen sources, which is useful in supporting cell survival (Stuart et al., 1999). As DEMs classified as amino acids were all found down-regulated in response to desiccation treatment, we infer that *Salmonella* utilized the sources generated by amino acid decomposition to maintain their metabolic activity in the early desiccation stage. Additionally, glycine, serine, and threonine metabolism was identified as a significantly different metabolic pathway by enrichment analysis (Figure 3A). Amino acids such as glycine, serine, and threonine may act as an important source of ATP production by entering the TCA cycle through pyruvate and acetyl-CoA (Ganesan et al., 2007). Therefore, in adverse desiccation environments, the decomposition of amino acids may promote ATP production and provide energy for the bacteria. Besides, the metabolomic analysis also identified intermediates such as glycine, betaine, choline, and glycerol, that are capable of regulating osmotic pressure in cells’ response to adverse conditions (Chen and Murata, 2002; Lebre et al., 2017).

Nucleic acids play an important role in maintaining the survival of organisms. After the desiccation treatment, the content of nucleotide derivatives of *S. Enteritidis* showed a downward trend, indicating that bacteria suffered DNA damage and nucleic acid degradation. The decrease in nucleotide metabolism (i.e., purine metabolism and pyrimidine metabolism, Figure 3A) might have affected the synthesis of DNA and RNA (Goncheva et al., 2022). Moreover, it has been reported that *Salmonella* could induce RNA molecule degradation in low water activity environments to obtain potential nutrients adaptable to the adverse environmental conditions (Burgess et al., 2016).

Additional significantly different metabolic pathways induced by desiccation treatment include pentose phosphate pathway (HMP) and vitamin B6 metabolism. HMP plays an important role in providing cells with intermediates of amino acids, vitamins, nucleotides, and is a major source of NADPH (Sprenger, 1995). Consistent with our results, in *Cronobacter sakazakii*, genes involved in the HMP are highly up-regulated during desiccation survival (Srikumar et al., 2019). When exposed to desiccation stress, *Salmonella* accumulates reactive oxygen species (ROS), which has deleterious consequences on the macromolecules (Christodoulou et al., 2018). It has been reported that enhanced HMP and increased production of NADPH could protect cells against desiccation-induced oxidative stress by reducing ROS levels (Boada et al., 2000). Vitamin B6 metabolism may also serve as a protective factor against desiccation-induced oxidative stress. As an antioxidant molecule, vitamin B6 has been implicated in defense against cellular oxidative stress in *Saccharomyces cerevisiae* (Chumnantana et al., 2005). It has also been reported that genes related to vitamin B6 pathway were involved in the response to 24 h-desiccation treatment in *Lactobacillus paracasei* (Palud et al., 2020).

### 3.7. Metabolic responses of *Salmonella enteric* induced by SMP storage

The 3-month samples were compared versus the 24 h samples to identify DEMs induced by SMP storage. A large amount of the DEMs was classified as amino acids (*n* = 18) and peptide (*n* = 6). The key pathways identified by enrichment analysis were also involved with amino acid metabolism (Figure 3B), suggesting that the metabolism of amino acids is prominently activated during *Salmonella*’s adaptation to long-term SMP storage. Multiple research studies have shown that genes related to amino acid metabolism are significantly induced under environmental stresses (Shah et al., 2013; Wu et al., 2021). The decreasing β-alanine metabolism pathway showed the most considerable impact on altered metabolic activity of *S. Enteritidis* during long-term storage in SMP (Figure 3B). A similar decreasing trend of alanine, aspartate, and glutamate metabolism was observed in metabolic response of *Escherichia coli* to ciprofloxacin stress (Li W. et al., 2018).

Water deficiency may cause changes in cell membrane components, such as phospholipids. The results of this study show that most of the detected phospholipids were up-regulated, indicating that the change of phospholipid content was an important metabolic response of *S. Enteritidis* during desiccation storage in SMP. *Salmonella* has been proven to cause changes in fatty acid composition under acid and cold stress (Álvarez-Ordóñez et al., 2009). Moreover, it has been reported that changes in phospholipid composition have detrimental effects on cellular envelope structure and function, and bacterial fitness in *Escherichia coli* (Rowlett et al., 2017), suggesting that adaptability of the *Salmonella* cells at 3-month storage might have been impaired due to desiccation stress. The glyceride metabolism, which is a main source of phospholipids, was identified as a significantly altered metabolic pathway (Figure 3B). The increased glyceride metabolism may potentially compensate for fat deficiency in SMP. In addition, all DEMs of fatty acid were found down-regulated in the metabolic analysis, which suggested that the catabolism of fatty acid may have served as a source of energy in low-moisture environments (Finn et al., 2013; Olaimat et al., 2020). Earlier transcriptome studies have shown that *Salmonella* is capable of inducing gene expressions related to fatty acid catabolism under drying stress, which has a non-negligible role in bacteria’s long-term adaptation to desiccation environments (Li et al., 2012).

DEMs classified as organic acid and carbohydrate were found mostly decreased in the desiccation-adapted *Salmonella* cells, which might have affected the normal progress of carbon circulation, and subsequently led to a reduction in carbon content and energy (Fernie et al., 2004). It is suggested that glycolysis has a noticeable effect on promoting carbon flow and supplying ATP under unfavorable conditions (Feng et al., 2021). Our results demonstrated a statistically significant metabolic pathway of glycolysis with up-regulated metabolites (Figure 3B), which may have played a significant role in *Salmonella*’s response to desiccation stress by providing necessary energy substances to support the key physiological cell activities during the long-term SMP storage.

In contrast to desiccation treatment, most DEMs related to nucleotide metabolism were found increased after SMP storage. This indicated that in the early stage under desiccation stress, nucleic acid molecules were the primary components to encounter injury during drying, and DNA repair mechanism would to be initiated following the adaptive stages of desiccation storage. The significant role of DNA repair pathways for desiccation tolerance has been emphasized in a transposon sequencing (Tn-seq) study of *Streptococcus pneumoniae*, in which the nucleotide excision repair pathway was identified as the most enriched category (Matthews et al., 2021). These findings demonstrate the importance of maintaining genome integrity in metabolic responses induced by desiccation stress.

### 3.8. Key enzyme activities and ATP content under desiccation stress

**Figure 4:**
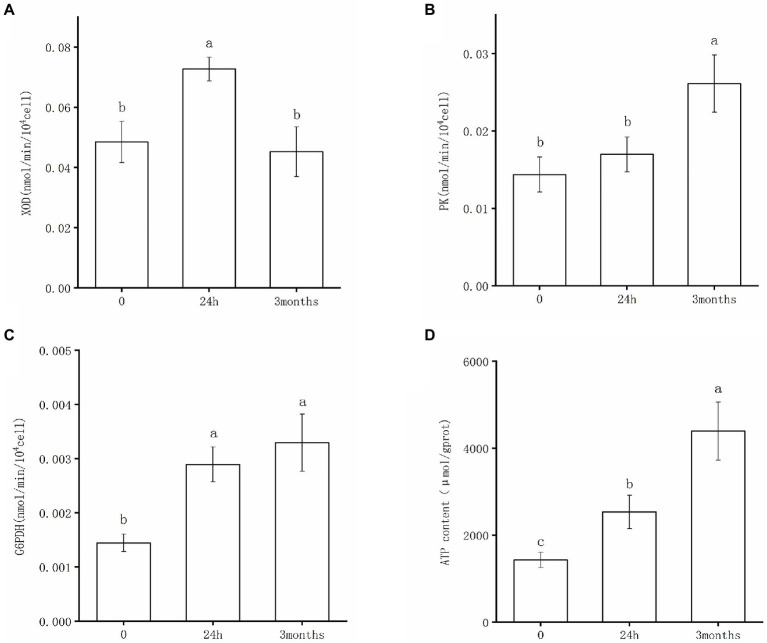
Enzyme activities of **(A)** XOD, **(B)** PK, **(C)** G6PDH, and changes of ATP content **(D)** after desiccation treatment and SMP storage. Different letters annotate significant difference in mean values ± standard deviations obtained from three independent tests (*p* < 0.05).

The enzyme activities of XOD, PK, and G6PDH of *Salmonella* following desiccation treatment and SMP storage are shown in Figure 4. XOD catalyzes the oxidation of hypoxanthine to xanthine. It has been implicated as a key oxidative enzyme to produce superoxide radicals and hydrogen peroxide molecules, which could lead to oxidative stress damage in bacteria cells (Wang et al., 2016). The results of the XOD activity test confirmed that *Salmonella* underwent nucleic acid degradation under desiccation condition. As shown in Figure 4A, XOD presented a distinctly high activity after 24 h-dehydration treatment; consistently, hypoxanthine was found down-regulated in metabolic analysis of the 24 h samples (Supplementary Table 1). The XOD activity significantly decreased and returned to its initial level in the 3-month samples, indicating that the degradation of nucleic acids occurred in the early stage of desiccation, whereas DNA repair pathways were initiated following the adaptive stage of desiccation storage. PK is the rate-limiting enzyme of the glycolysis pathway and is one of the key enzymes involved in ATP production (Guo et al., 2021). In the initial stage of desiccation, as the metabolic response of *Salmonella* was dominated by molecular damage and biosynthesis promotion, there was no significant difference in the activity of PK between the 0-samples and 24 h samples (Figure 4B). After 3-month storage in SMP, the PK activity greatly increased (Figure 4B), which proved that glycolysis pathway exerted a crucial role in *Salmonella*’s persistence during the drying adaptation. As one of the main enzymes in the pentose phosphate pathway, G6PDH retained greater activity after desiccation treatment (Figure 4C). It appeared that G6PDH was a key factor for *Salmonella’*s adaptation in the low moisture environment. With the activated G6PDH activity, the increased production of NADPH was useful in maintaining the balance of biosynthesis and antioxidant capacity of the bacteria, which might be the key to desiccation stress regulation.

Additionally, the intracellular ATP content was investigated to validate the metabolic response of *S. Enteritidis* under desiccation stress. It can be observed that the ATP content significantly increased after the initial desiccation treatment and long-term SMP storage, respectively (Figure 4D). This indicated that *Salmonella* continuously produced ATP to obtain energy for cell biosynthesis and executed functions necessary to maintain normal organism operation when exposed to desiccation stress. In consistent with our results, a recent study has reported that genes coding ATP synthase played an important role in desiccation survival of *S. Typhimurium*; interestingly, this study screened a transposon mutant library of *S. Typhimurium* to identify desiccation survival genes and found these genes were mostly related to energy production and conversion (Mandal and Kwon, 2017).

## 4. Conclusion

Non-targeted metabolomics was performed by GC–MS and LC–MS/MS approaches to investigate the adaptive response of *Salmonella* under desiccation stress and SMP storage. Metabolic responses of *S. Enteritidis* induced by 24 h-desiccation treatment mainly involved carbon/nitrogen supply from amino acid catabolism, supply of potential nutrients by nucleotide degradation, and antioxidant protection against desiccation-induced oxidative stress. As for SMP storage, the metabolite changes and regulatory responses appeared to be more complicated due to the influence of food ingredients and storage time. The potential adaptive mechanisms of *Salmonella* in long-term desiccation storage include: the promotion of amino acid metabolism to maintain cell stability, up-regulation of glycerolipid metabolism to maintain the integrity of cell membrane, catabolism of fatty acids to produce energy, and enhancement of carbon flow and ATP production through glycolytic pathway. The analyses of enzyme activities of XOD, PK, and G6PDH and ATP content provided further evidence that supported the metabolic responses revealed by non-targeted metabolomics. The results of this study manifested the differences in desiccation-induced metabolic responses of *Salmonella* between the initial stage of desiccation stress and the following long-term adaptive stage, which would be beneficial for the development of targeted measures to reduce food safety risk from desiccation-adapted *Salmonella* in the production and long-term storage of LMFs.

## Data availability statement

The original contributions presented in the study are included in the article/Supplementary material, further inquiries can be directed to the corresponding author.

## Author contributions

SL: methodology, writing-review, and editing. YC: methodology and writing-original draft. JZ: writing-review and editing, and software. HYZ: writing-review. ZM, SC, YY, and WX: writing-review and editing. HMZ: funding acquisition and methodology, project administration, supervision, and writing-review and editing. All authors contributed to the article and approved the submitted version.

## Funding

This study was supported by the National Natural Science Foundation of China (Nos. 31972044 and 32202186).

## Conflict of interest

The authors declare that the research was conducted in the absence of any commercial or financial relationships that could be construed as a potential conflict of interest.

## Publisher’s note

All claims expressed in this article are solely those of the authors and do not necessarily represent those of their affiliated organizations, or those of the publisher, the editors and the reviewers. Any product that may be evaluated in this article, or claim that may be made by its manufacturer, is not guaranteed or endorsed by the publisher.

## Footnotes

^1^ https://bioconductor.org/packages/release/bioc/html/xcms.html

^2^ https://hmdb.ca/

^3^ https://www.kegg.jp/

^4^ https://www.r-project.org/

## Supplementary material

SUPPLEMENTARY TABLE The list of differentially expressed metabolites.

SUPPLEMENTARY FIGURE S1 QC results of GC-TOF-MS. (A) Overlapping TICs of duplicate samples; (B) TIC of blank sample.

SUPPLEMENTARY FIGURE S2 TICs in NEG mode of all samples by UHPLC-QE-MS.

SUPPLEMENTARY FIGURE S3 TICs in POS mode of all samples by UHPLC-QE-MS.

SUPPLEMENTARY FIGURE S4 Results of OPLS-DA. (A) score scatter plot and (C) permutation test of OPLS-DA model for 0-samples and 24h samples; (B) score scatter plot and (D) permutation test of the OPLS-DA model for 24h samples and 3-month samples. The abscissa t[1]P represents the predicted principal component score of PC1, which explains the difference between the two samples. The ordinate t[1]O represents the score of the orthogonal principal component, which explains the difference within the samples.

SUPPLEMENTARY FIGURE S5 Volcano plot of differential expressed metabolites induced by (A) desiccation treatment and (B) SMP storage.

SUPPLEMENTARY FIGURE S6 Heatmap of hierarchical clustering analysis of all the identified differentially expressed metabolites in the 0-, 24h and 3-month samples.
