# Supplementary material for: Metabolomics-based response of Salmonella to desiccation stress and skimmed milk powder storage
Source: Front Microbiol. 2023 Feb 23;14:1092435. doi: 10.3389/fmicb.2023.1092435 (PMC9996163; doi:10.3389/fmicb.2023.1092435)
Supplement: SUPPLEMENTARY TABLE — The list of differentially expressed metabolites. [file Table_1.DOCX]

**Supplementary Table 1**. Differentially expressed metabolites induced by 24h-desiccation treatment

| DEMs | VIP | P-value | FC | Trend | Classification | KEGG annotation |
| --- | --- | --- | --- | --- | --- | --- |
| DL-glutamic acid | 1.78 | 0.02 | 0.19 | ↓ | Amino acid | － |
| L-threonine | 1.77 | 3.23 ×10^-3^ | 0.26 | ↓ | Amino acid | ABC transporter; Metabolic pathway |
| Serine | 1.52 | 0.05 | 0.67 | ↓ | Amino acid | － |
| Hypoxanthine | 1.85 | 0.04 | 0.22 | ↓ | Nucleic acid | Purine metabolism |
| Adenine (A) | 1.62 | 0.05 | 0.67 | ↓ | Nucleic acid | Purine metabolism |
| Cytosine (C) | 1.64 | 0.04 | 0.42 | ↓ | Nucleic acid | pyrimidine metabolism |
| Deoxyguanosine monophosphate (dGMP) | 1.82 | 5.12×10^-6^ | 0.03 | ↓ | Nucleic acid | Purine metabolism |
| Uridine acid (UMP) | 1.85 | 6.49×10^-6^ | 0.05 | ↓ | Nucleic acid | Pyrimidine metabolism; Biosynthesis of cofactors |
| Pyridoxine | 1.72 | 5.72 ×10^-3^ | 3.25 | ↑ | Vitamins and cofactors | Biosynthesis of cofactors |
| L-gulose | 1.54 | 0.03 | 0.39 | ↓ | Carbohydrate | － |
| Gluconic acid | 1.59 | 0.03 | 1.21 | ↑ | Carbohydrate | Metabolic pathway |
| Imidazole acetic acid | 1.67 | 0.01 | 0.36 | ↓ | Organic acid | Histidine metabolism |
| 3-hydroxybutyric acid | 1.80 | 2.74×10^-3^ | 0.16 | ↓ | Organic acid | － |
| L-3-phenyllactic acid | 1.81 | 0.01 | 0.27 | ↓ | Organic acid | Phenylalanine metabolism |
| 3-hydroxyphenylacetic acid | 1.59 | 0.03 | 1.40 | ↑ | Organic acid | Phenylalanine metabolism |
| 2- (methylamino) benzoic acid | 1.61 | 0.05 | 1.65 | ↑ | Organic acid | － |
| P-anisol | 1.84 | 1.50×10^-3^ | 4.36 | ↑ | Alcohols | － |
| Vegetable alcohol | 1.83 | 2.16×10^-3^ | 1.88 | ↑ | Alcohols | － |
| Dodecanol | 1.81 | 0.03 | 1.37 | ↑ | Alcohols | － |
| Hexadecyl alcohol | 1.60 | 0.04 | 1.67 | ↑ | Alcohols | － |
| Pyrophosphate (PPI) | 1.73 | 0.01 | 4.84 | ↑ | Inorganic salt | Oxidative phosphorylation |
| Phosphate (PI) | 1.86 | 0.02 | 52.10 | ↑ | Inorganic salt | Oxidative phosphorylation；  ABC transporter |
| linoleic acid | 1.85 | 2.0×10^-4^ | 6.76 | ↑ | Fatty acid | Biosynthesis of unsaturated fatty acids |
| stearic acid | 1.67 | 0.03 | 1.96 | ↑ | Fatty acid | biosynthesis of fatty acid;  Biosynthesis of unsaturated fatty acids |
| 4-acetylbutyric acid 2 | 1.71 | 0.02 | 1.46 | ↑ | Fatty acid | － |
| Palmitoleic acid | 1.83 | 2.30×10^-3^ | 0.49 | ↓ | Fatty acid | biosynthesis of fatty acid |
| Plant sphingosine | 1.62 | 0.04 | 0.60 | ↓ | Lipid | Metabolic pathway |
| (13R, 14R) -8-label-13,14,15-triol | 1.74 | 0.03 | 0.24 | ↓ | Lipid | － |
| Caffeisterone | 1.88 | 7.22×10^-6^ | 0.10 | ↓ | Lipid | － |
| Tripterygium wilfordii f | 1.81 | 9.97×10^-4^ | 0.48 | ↓ | Lipid | － |
| 1-palmitonin | 1.65 | 0.03 | 0.66 | ↓ | Lipid | － |
| P-menthol-1,3,8-triene | 1.77 | 0.01 | 0.80 | ↓ | Lipid | － |
| 11B, 21-dihydroxy-5b- pregnane-3,20 Dione | 1.75 | 2.09×10^-6^ | 0.04 | ↓ | Lipid | － |
| Lysophosphatidylcholine (14:1 (9z)) | 1.67 | 0.04 | 0.27 | ↓ | Lipid | － |
| Phosphatidylethanolamine (20:3 (8Z, 11z, 14z) /14:1 (9z)) | 1.48 | 0.01 | 0.35 | ↓ | Lipid | － |
| Phosphatidylethanolamine (22:2 (13z, 16Z) /14:0) | 1.62 | 0.02 | 0.34 | ↓ | Lipid | － |
| Phosphatidylethanolamine (20:1 (11z) /15:0) | 1.68 | 0.01 | 0.31 | ↓ | Lipid | － |
| Phosphatidylethanolamine (20:0/18:2 (9z, 12z)) | 1.73 | 0.02 | 0.30 | ↓ | Lipid | － |
| Phosphatidylethanolamine (15:0/18:1 (11z)) | 1.73 | 0.04 | 0.27 | ↓ | Lipid | － |
| uric acid | 1.48 | 0.03 | 0.52 | ↓ | Other | Histidine metabolism |
| 2-methyl-3 - (2-propenyl) pyrazine | 1.73 | 0.03 | 0.23 | ↓ | Other | － |
| 3α- 4,5,7-tetrahydro-5-hydroxy-1h-isoindole-1,3-dione | 1.61 | 0.02 | 0.44 | ↓ | Other | － |
| 2-amino-3,8-dimethyl-3h-imidazo [4,5-f] quinoxaline | 1.71 | 0.03 | 0.28 | ↓ | Other | － |
| Propyl 1- (propyl sulfuroyl) propyl disulfide | 1.63 | 0.05 | 2.48 | ↑ | Other | － |
| Phenylpropionic acid | 1.87 | 2.74×10^-4^ | 0.49 | ↓ | Other | － |
| L-cis-3-amino-2-pyrrolidine carboxylic acid | 1.72 | 0.01 | 0.53 | ↓ | Other | － |
| Acetylhydrazine | 1.59 | 0.01 | 2.03 | ↑ | Other | － |
| 2,4,5-trimethoxybenzaldehyde | 1.69 | 0.05 | 0.20 | ↓ | Other | － |
| Ketoacids | 1.88 | 0.01 | 3.57 | ↑ | Other | － |
| M-chlorohippuric acid | 1.64 | 0.01 | 0.46 | ↓ | Other | － |
| Concanavalin | 1.48 | 0.05 | 0.52 | ↓ | Other | － |
| Cimicifugin a | 1.85 | 3.40×10^-3^ | 0.19 | ↓ | Other | － |
| 4-trimethylaminobutyric acid(γ- Butyryl betaine) | 1.74 | 0.03 | 0.32 | ↓ | Other | － |
| 2,3-dihydroxypyridine | 1.89 | 2.01×10^-3^ | 11.07 | ↑ | Other | － |
| Lactone triacetate | 1.68 | 0.03 | 1.52 | ↑ | Other | － |
| 2,2-dimethylsuccinic acid | 1.57 | 0.04 | 1.16 | ↑ | Other | － |
| Atrazine-2-hydroxy-5 | 1.77 | 0.01 | 1.58 | ↑ | Other | － |
| Biuret | 1.71 | 0.02 | 1.29 | ↑ | Other | － |

**Supplementary Table 2.** Differentially expressed metabolites induced by 3-month storage in SMP

| DEMs | VIP | P-value | FC | Trend | Classification | KEGG annotation |
| --- | --- | --- | --- | --- | --- | --- |
| γ-Aminobutyric acid (GABA) | 1.55 | 5.08×10^-5^ | 0.24 | ↓ | Amino acid | Arginine and proline metabolism;  β- Alanine metabolism;  Butyric acid metabolism;  Group induction;  Nicotinic acid and nicotinamide metabolism |
| L-cis-3-aminoproline | 1.41 | 0.03 | 0.62 | ↓ | Amino acid | － |
| 5-hydroxy-L-tryptophan | 1.44 | 4.47×10^-3^ | 0.39 | ↓ | Amino acid | － |
| N-a-acetyl-l-arginine | 1.47 | 1.20×10^-3^ | 0.36 | ↓ | Amino acid | － |
| L-threonine | 1.41 | 0.04 | 0.57 | ↓ | Amino acid | Glycine, serine and threonine metabolism;  Biosynthesis of valine, leucine and isoleucine;  ABC transporter;  Aminoacyl tRNA biosynthesis;  Biosynthesis of single lactam |
| Alanine 3-sulfite | 1.47 | 0.01 | 0.76 | ↓ | Amino acid | Cysteine and methionine metabolism |
| L-serine | 1.30 | 0.03 | 0.21 | ↓ | Amino acid | － |
| Leucine | 1.35 | 0.04 | 0.49 | ↓ | Amino acid | － |
| β- alanine | 1.12 | 0.05 | 0.26 | ↓ | Amino acid | Pyrimidine metabolism;  β-Alanine metabolism;  Biosynthesis of cofactors |
| L-Leucine | 1.21 | 0.05 | 0.50 | ↓ | Amino acid | － |
| L-glutamic acid | 1.39 | 7.77×10^-4^ | 4.20 | ↑ | Amino acid | Cyano amino acid metabolism |
| N-acetylhistidine | 1.50 | 3.73×10^-3^ | 1.67 | ↑ | Amino acid | － |
| Citrulline | 1.51 | 0.01 | 4.44 | ↑ | Amino acid | Biosynthesis of arginine |
| L-arginine | 1.43 | 0.02 | 1.74 | ↑ | Amino acid | Arginine and proline metabolism;  Aminoacyl tRNA biosynthesis;  Arginine biosynthesis;  Biosynthesis of single lactam;  ABC transporter |
| Methylaminoglutamic acid | 1.29 | 0.04 | 1.59 | ↑ | Amino acid | － |
| N-acetylserine | 1.48 | 2.97×10^-4^ | 4.48 | ↑ | Amino acid | － |
| L-homoarginine | 1.36 | 0.02 | 2.61 | ↑ | Amino acid | － |
| 2-azacyclobutanecarboxylic acid | 1.38 | 0.03 | 1.90 | ↑ | Amino acid | － |
| Adenosine 2'- phosphate | 1.51 | 0.01 | 7.11 | ↑ | Nucleic acid | － |
| Guanine G | 1.55 | 2.37×10^-3^ | 1.77 | ↑ | Nucleic acid | Purine metabolism |
| Deoxyguanosine monophosphate (dGMP) | 1.49 | 0.03 | 15.97 | ↑ | Nucleic acid | Purine metabolism |
| 5'- uridine monophosphate (UMP) | 1.53 | 0.03 | 13.81 | ↑ | Nucleic acid | Pyrimidine metabolism;  Biosynthesis of cofactors |
| Thymine t | 1.52 | 6.01×10^-4^ | 0.59 | ↓ | Nucleic acid | Pyrimidine metabolism |
| 2-phosphate-d-glyceric acid | 1.51 | 0.01 | 11.99 | ↑ | Organic acid | Methane metabolism;  Glyceride metabolism;  Glycine, serine and threonine metabolism |
| P-dodecylbenzene sulfonic acid | 1.36 | 0.04 | 0.72 | ↓ | Organic acid | － |
| 2-oxoisohexanoic acid | 1.58 | 2.27×10^-5^ | 0.21 | ↓ | Organic acid | － |
| Maleic acid | 1.43 | 0.01 | 0.17 | ↓ | Organic acid | Butyric acid metabolism;  Nicotinic acid and nicotinamide metabolism |
| Ketoglutarate | 1.35 | 0.02 | 0.25 | ↓ | Organic acid | － |
| Tartaric acid | 1.54 | 0.01 | 0.49 | ↓ | Organic acid | － |
| Methyl succinic acid | 1.47 | 0.01 | 0.69 | ↓ | Organic acid | － |
| 2,2-dimethylsuccinic acid | 1.57 | 3.65×10^-4^ | 0.59 | ↓ | Organic acid | － |
| 4-acetylbutyric acid 2 | 1.34 | 0.03 | 0.53 | ↓ | Organic acid | － |
| D-arabinol | 1.49 | 3.25×10^-3^ | 0.48 | ↓ | Carbohydrate | － |
| 1-deoxy-d-glucose alcohol | 1.47 | 0.01 | 0.17 | ↓ | Carbohydrate | － |
| Dihydroxyacetone | 1.58 | 0.01 | 0.01 | ↓ | Carbohydrate | Methane metabolism;  Glyceride metabolism; |
| phosphoric acid | 1.43 | 0.01 | 7.43 | ↑ | Inorganic salt | ABC transporter;  Quorum sensing |
| Hydrogen phosphate | 1.49 | 0.01 | 2.58 | ↑ | Inorganic salt | － |
| Pyridoxine | 1.57 | 0.01 | 0.14 | ↓ | Vitamins and cofactors | Biosynthesis of cofactors |
| Triethylamine | 1.31 | 0.05 | 0.58 | ↓ | Amine | － |
| Aspartate leucine | 1.52 | 1.01×10^-3^ | 3.83 | ↑ | Peptide | － |
| Glutamyl phenylalanine | 1.34 | 7.33×10^-4^ | 7.60 | ↑ | Peptide | － |
| γ-glutamine alanine | 1.54 | 2.01×10^-3^ | 1.81 | ↑ | Peptide | － |
| Propionylglycine | 1.56 | 0.001 | 3.04 | ↑ | Peptide | － |
| Valine aspartic acid | 1.45 | 0.02 | 4.76 | ↑ | Peptide | － |
| Isoleucine alanine | 1.56 | 0.03 | 5.95 | ↑ | Peptide | － |
| Nicotine | 1.53 | 1.62×10^-3^ | 0.70 | ↓ | Alkaloid | － |
| Berberine | 1.48 | 0.01 | 0.59 | ↓ | Alkaloid | － |
| (r)-cinnamic base | 1.55 | 0.01 | 0.66 | ↓ | Alkaloid | － |
| Xanthoside | 1.41 | 0.03 | 0.69 | ↓ | Alkaloid | Purine metabolism;  ABC transporter |
| α-crotonic acid | 1.44 | 0.02 | 0.62 | ↓ | Fatty acid | － |
| Apigenic acid | 1.28 | 0.04 | 0.54 | ↓ | Fatty acid | － |
| Tridecanoic acid | 1.47 | 0.01 | 0.70 | ↓ | Fatty acid | － |
| Linoleic acid | 1.40 | 0.03 | 0.75 | ↓ | Fatty acid | － |
| 2-hydroxystearic acid | 1.53 | 6.12×10^-4^ | 0.45 | ↓ | Fatty acid | － |
| Heptanic acid | 1.32 | 0.04 | 0.83 | ↓ | Fatty acid | － |
| Nonanoic acid | 1.42 | 0.01 | 0.50 | ↓ | Fatty acid | － |
| Lauramide DEA | 1.45 | 0.01 | 0.68 | ↓ | Lipid | － |
| Trans hexadecyl-2-enol carnitine | 1.36 | 0.04 | 0.73 | ↓ | Lipid | － |
| Jasmonic acid | 1.32 | 0.05 | 0.66 | ↓ | Lipid | － |
| Cetane | 1.58 | 1.53×10^-3^ | 0.02 | ↓ | Lipid | － |
| Thymol | 1.58 | 2.88×10^-3^ | 0.09 | ↓ | Lipid | － |
| Lysophosphatidylethanolamine (16:0/0:0) | 1.47 | 0.01 | 4.89 | ↑ | Lipid | － |
| (13r, 14r) -8-label-13,14,15-triol | 1.51 | 7.10×10^-4^ | 4.00 | ↑ | Lipid | － |
| Caffeisterone | 1.54 | 0.03 | 4.94 | ↑ | Lipid | － |
| Phosphatidylic acid (16:1 (9z) /16:0) | 1.38 | 0.02 | 2.11 | ↑ | Lipid | － |
| Lysophosphatide (18:1 (9z) /0:0) | 1.38 | 0.02 | 9.84 | ↑ | Lipid | － |
| Tripterygium wilfordii f | 1.54 | 0.04 | 4.49 | ↑ | Lipid | － |
| 11B, 21 dihydroxy 5B pregnane 3,20 Dione | 1.46 | 1.50×10^-5^ | 19.19 | ↑ | Lipid | － |
| Lysophosphatide (14:1 (9z)) | 1.45 | 0.01 | 3.90 | ↑ | Lipid | － |
| Monkstatin | 1.42 | 0.03 | 2.36 | ↑ | Lipid | － |
| Cimicifugin B | 1.50 | 2.70×10^-3^ | 2.56 | ↑ | Lipid | － |
| Cimicifugin C | 1.40 | 0.01 | 2.76 | ↑ | Lipid | － |
| Phosphatidyl acetamide (15:0/16:1 (9z)) | 1.48 | 2.12×10^-3^ | 3.40 | ↑ | Lipid | － |
| Phosphatidyl acetamide (14:0/16:1 (9z)) | 1.47 | 0.01 | 3.11 | ↑ | Lipid | － |
| Phosphatidyl acetamide (15:0/15:0) | 1.50 | 2.61×10^-3^ | 2.86 | ↑ | Lipid | － |
| Phosphatidyl acetamide (14:1 (9z) /18:0) | 1.50 | 3.08×10^-3^ | 3.69 | ↑ | Lipid | － |
| Phosphatidylcholine (20:1 (11z) /14:1 (9z)) | 1.54 | 3.06×10^-3^ | 2.62 | ↑ | Lipid | － |
| Phosphatidyl acetamide (20:2 (11z, 14z) /15:0) | 1.40 | 0.02 | 3.38 | ↑ | Lipid | － |
| Phosphatidyl acetamide (20:1 (11z) /15:0) | 1.48 | 2.59×10^-3^ | 5.13 | ↑ | Lipid | － |
| Phosphatidyl acetamide (18:2 (9z, 12z) /15:0) | 1.35 | 3.87×10^-3^ | 5.17 | ↑ | Lipid | － |
| Phosphatidyl acetamide (20:0/18:2 (9z, 12z)) | 1.47 | 0.01 | 3.21 | ↑ | Lipid | － |
| Phosphatidyl acetamide (15:0/18:1 (11z)) | 1.49 | 0.01 | 3.52 | ↑ | Lipid | － |
| Phosphatidylcholine (16:0/p-18:1 (11z)) | 1.31 | 0.04 | 2.45 | ↑ | Lipid | － |
| L-methylpyrrolo [1,2-a] pyrazine | 1.55 | 0.05 | 0.12 | ↓ | Other | － |
| Creatinine | 1.47 | 0.01 | 0.52 | ↓ | Other | Arginine and proline metabolism |
| ε caprolactam | 1.41 | 0.04 | 0.52 | ↓ | Other | － |
| Oleamide | 1.55 | 1.29×10^-3^ | 2.63 | ↑ | Other | － |
| Phenylpropionic acid | 1.54 | 5.23×10^-4^ | 0.34 | ↓ | Other | － |
| Indole | 1.55 | 1.70×10^-3^ | 0.59 | ↓ | Other | － |
| Acetylhydrazine | 1.56 | 9.59×10^-4^ | 0.60 | ↓ | Other | － |
| M-coumaric acid | 1.50 | 1.97×10^-3^ | 0.29 | ↓ | Other | － |
| 5'- methylthioadenosine | 1.33 | 0.04 | 1.37 | ↑ | Other | Cysteine and methionine metabolism |
| 2-piperidone | 1.57 | 1.91×10^-4^ | 0.57 | ↓ | Other | － |
| Fapy adenine | 1.40 | 0.02 | 0.61 | ↓ | Other | － |
| 2-methyl-3 - (2-propenyl) pyrazine | 1.43 | 0.04 | 0.32 | ↓ | Other | － |
| 2-pyrrolidone | 1.51 | 0.01 | 0.27 | ↓ | Other | － |
| M-xylene | 1.37 | 0.03 | 0.82 | ↓ | Other | － |
| Aristotle ketone | 1.46 | 0.01 | 0.65 | ↓ | Other | － |
| L- (1-pyrrolyl) - 2-butanone | 1.31 | 0.04 | 0.76 | ↓ | Other | － |
| Acetone cyanohydrin | 1.42 | 0.03 | 0.62 | ↓ | Other | Biosynthesis of valine, leucine and isoleucine |
| (2r, 3r, 4r) - 2-amino-4-hydroxy-3-methylvaleric acid | 1.47 | 4.38×10^-3^ | 0.58 | ↓ | Other | － |
| Tromethamine | 1.35 | 0.04 | 0.78 | ↓ | Other | － |
| (2r) - 2-hydroxy-2-methylnitrile | 1.52 | 3.11×10^-3^ | 0.64 | ↓ | Other | Cyano amino acid metabolism |
| 2,4,5-trimethoxybenzaldehyde | 1.40 | 0.01 | 3.08 | ↑ | Other | － |
| 1- (hydroxymethyl) - 5,5-dimethyl-2,4-imidazoline Dione | 1.44 | 0.02 | 3.63 | ↑ | Other | － |
| Polyoxyethylene dibasic acid | 1.50 | 2.10×10^-3^ | 3.04 | ↑ | Other | － |
| 3α- 4,5,7-tetrahydro-5-hydroxy-1h-isoindole-1,3 (2h) - Dione | 1.40 | 0.03 | 0.28 | ↓ | Other | － |
| 1-methyl-1,3-cyclohexadiene | 1.55 | 9.63×10^-4^ | 0.69 | ↓ | Other | － |
| Hop ether | 1.48 | 0.01 | 0.57 | ↓ | Other | － |
| Concanavalin Degreaser | 1.21 | 0.02 | 0.14 | ↓ | Other | － |
| d-1-amino-2-pyrrolidine formic acid | 1.43 | 0.05 | 0.29 | ↓ | Other | － |
| 2- (methylamino) benzoic acid | 1.51 | 0.05 | 0.44 | ↓ | Other | － |
| Homocysteine thiolactone | 1.33 | 0.04 | 0.16 | ↓ | Other | － |
| Phenylpropiononitrile | 1.36 | 0.04 | 0.66 | ↓ | Other | － |
| Diethyl phthalate | 1.51 | 3.72×10^-3^ | 0.65 | ↓ | Other | － |
| Cumene | 1.51 | 2.06×10^-3^ | 0.68 | ↓ | Other | － |
| Demethylantipyrine | 1.24 | 0.03 | 0.43 | ↓ | Other | － |
| 2,3-dihydroxypyridine | 1.11 | 0.03 | 0.40 | ↓ | Other | － |
| Resorcinol | 1.06 | 0.04 | 0.39 | ↓ | Other | － |
| Uric acid | 1.10 | 0.03 | 3.92 | ↑ | Other | Purine metabolism |
