# Supplementary figures and images for: Metabolomics-based response of Salmonella to desiccation stress and skimmed milk powder storage
Source: Front Microbiol. 2023 Feb 23;14:1092435. doi: 10.3389/fmicb.2023.1092435 (PMC9996163; doi:10.3389/fmicb.2023.1092435)

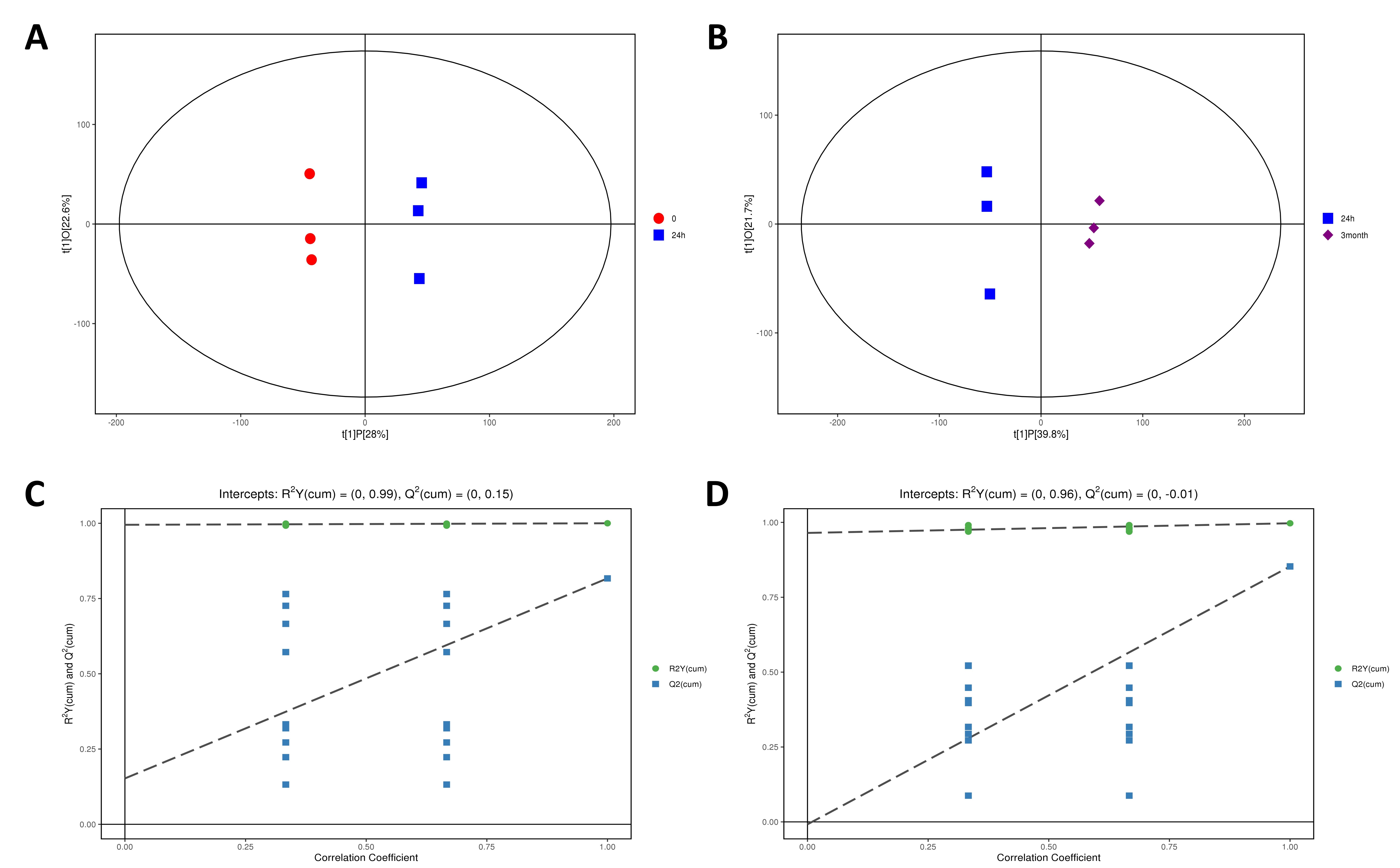

Supplement: SUPPLEMENTARY FIGURE S1 — QC results of GC-TOF-MS. (A) Overlapping TICs of duplicate samples; (B) TIC of blank sample. [file Image_1.JPEG]

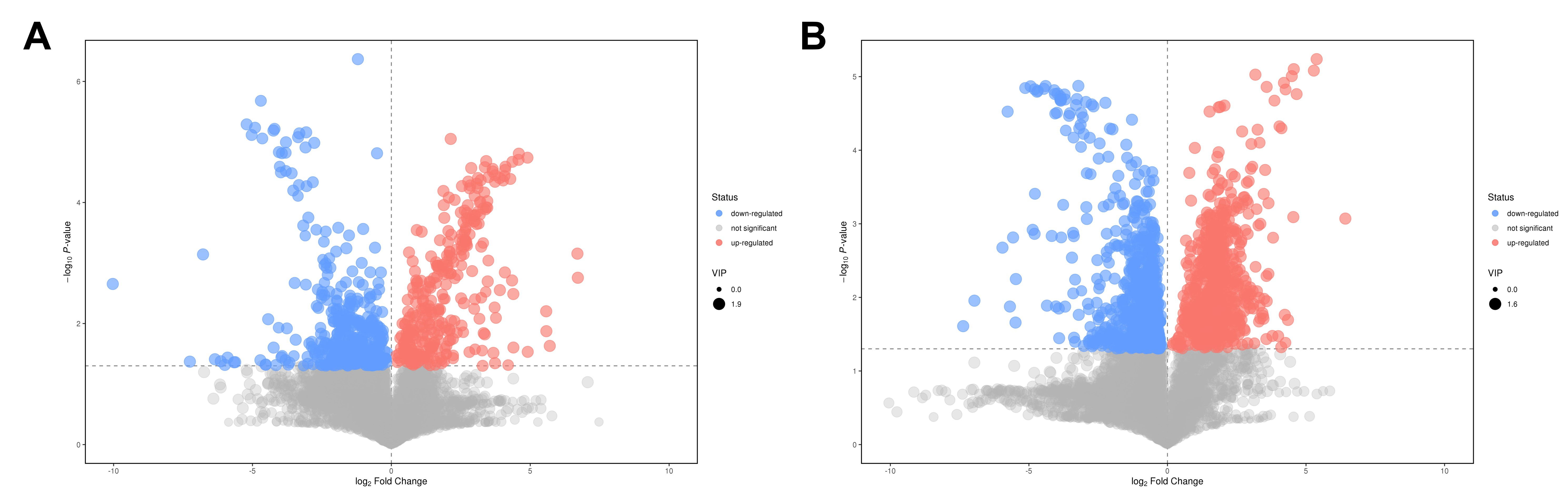

Supplement: SUPPLEMENTARY FIGURE S2 — TICs in NEG mode of all samples by UHPLC-QE-MS. [file Image_2.JPEG]

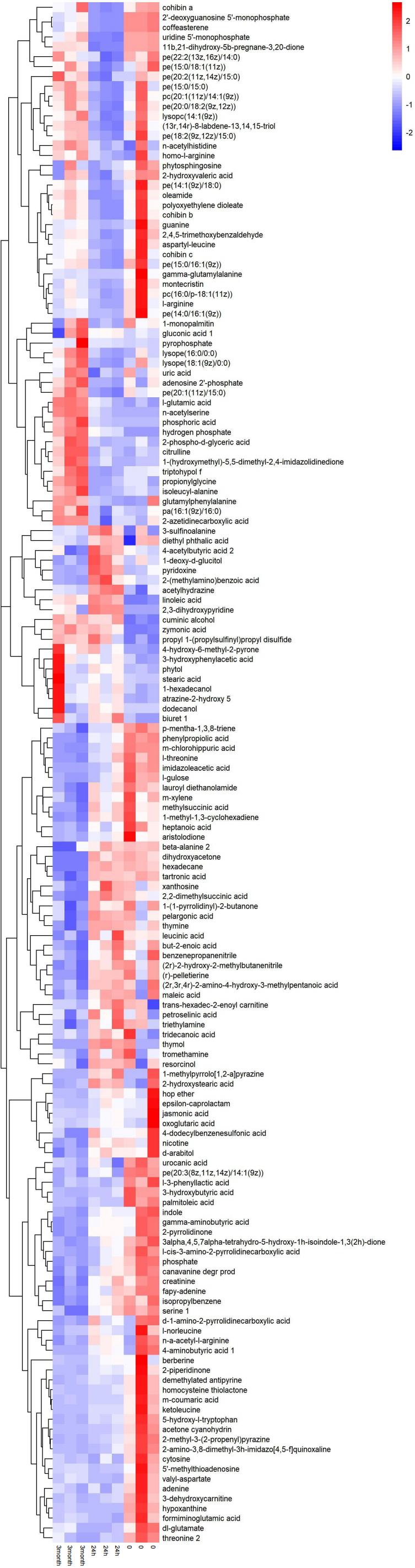

Supplement: SUPPLEMENTARY FIGURE S3 — TICs in POS mode of all samples by UHPLC-QE-MS. [file Image_3.JPEG]

**A**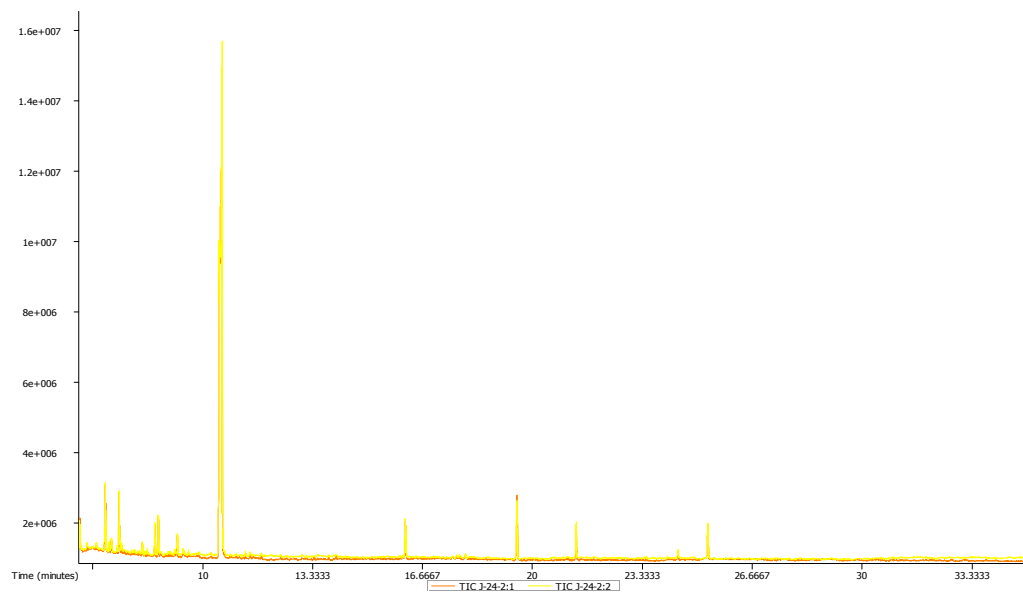**B**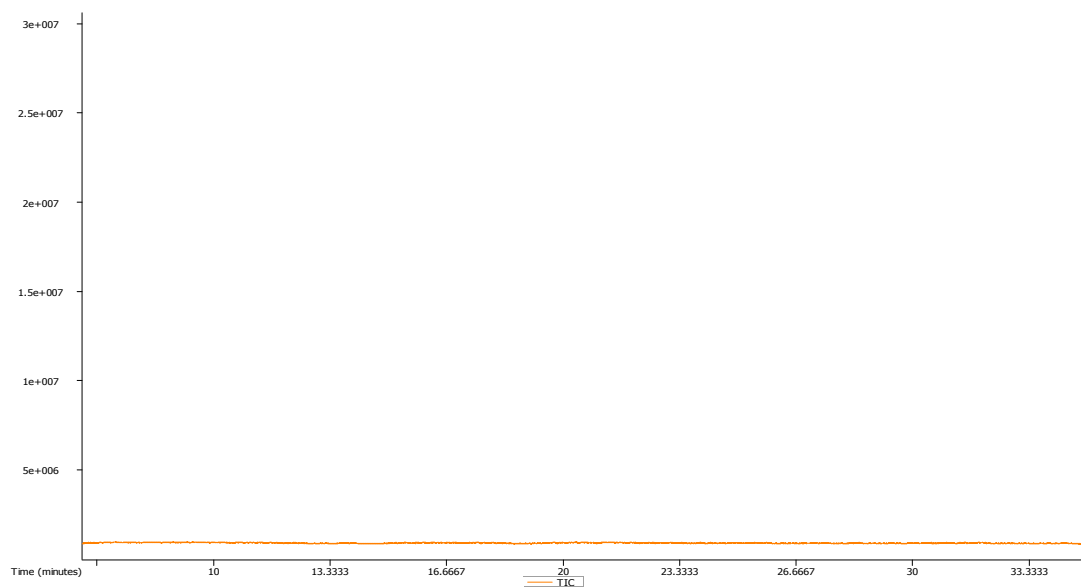

Supplement: SUPPLEMENTARY FIGURE S4 — Results of OPLS-DA. (A) score scatter plot and (C) permutation test of OPLS-DA model for 0-samples and 24h samples; (B) score scatter plot and (D) permutation test of the OPLS-DA model for 24h samples and 3-month samples. The abscissa t[1]P represents the predicted principal component score of PC1, which explains the difference between the two samples. The ordinate t[1]O represents the score of the orthogonal principal component, which explains the difference within the samples. [file Data_Sheet_1.PDF]

negJ\_0\_1

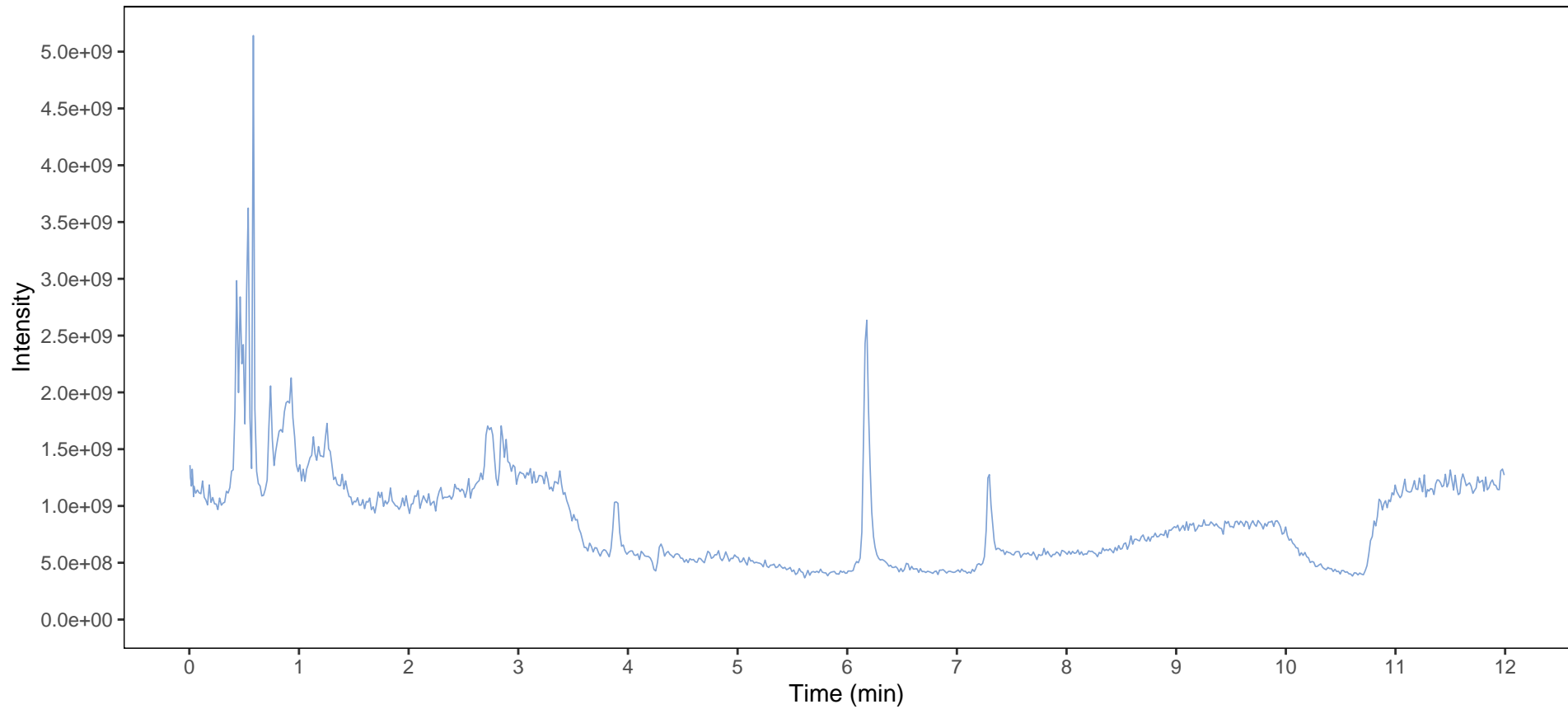

negJ\_0\_2

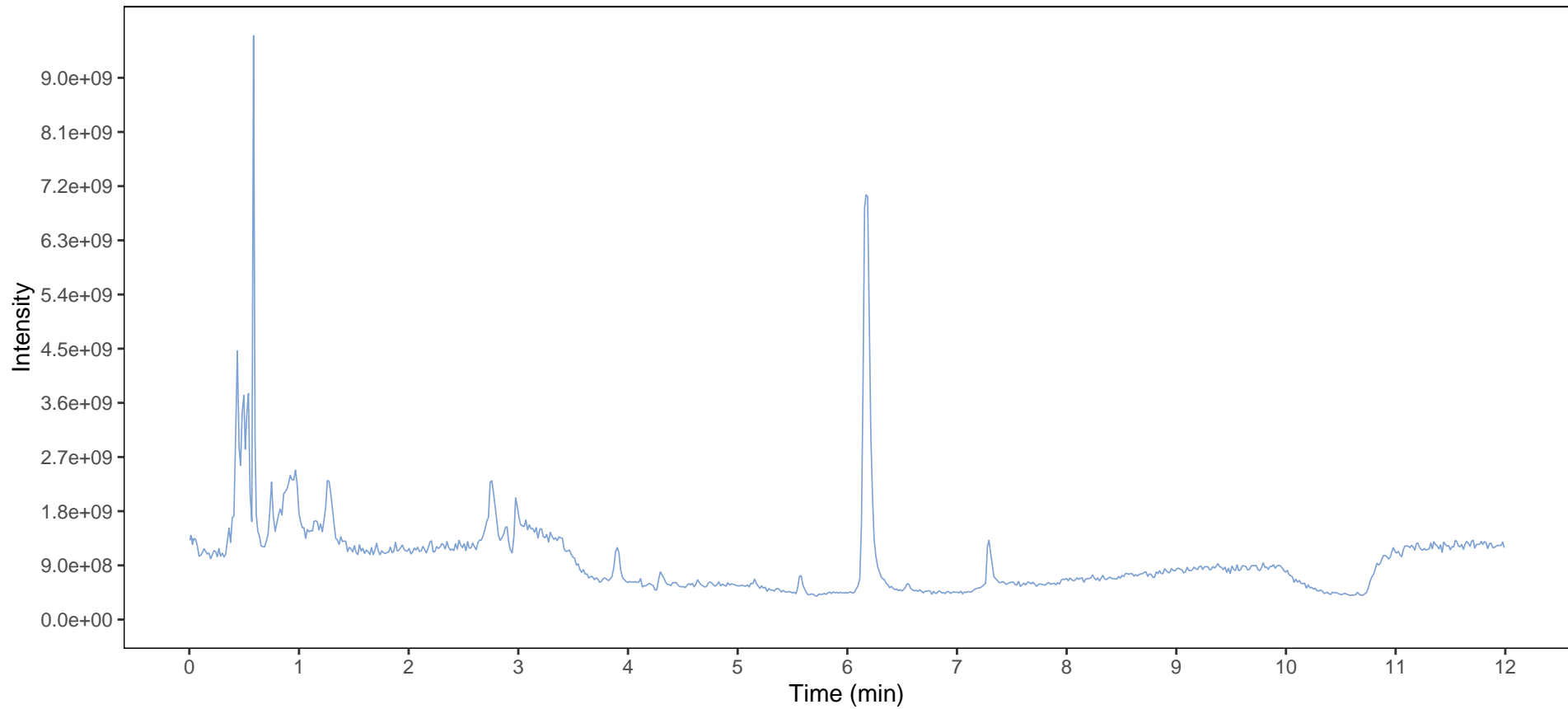

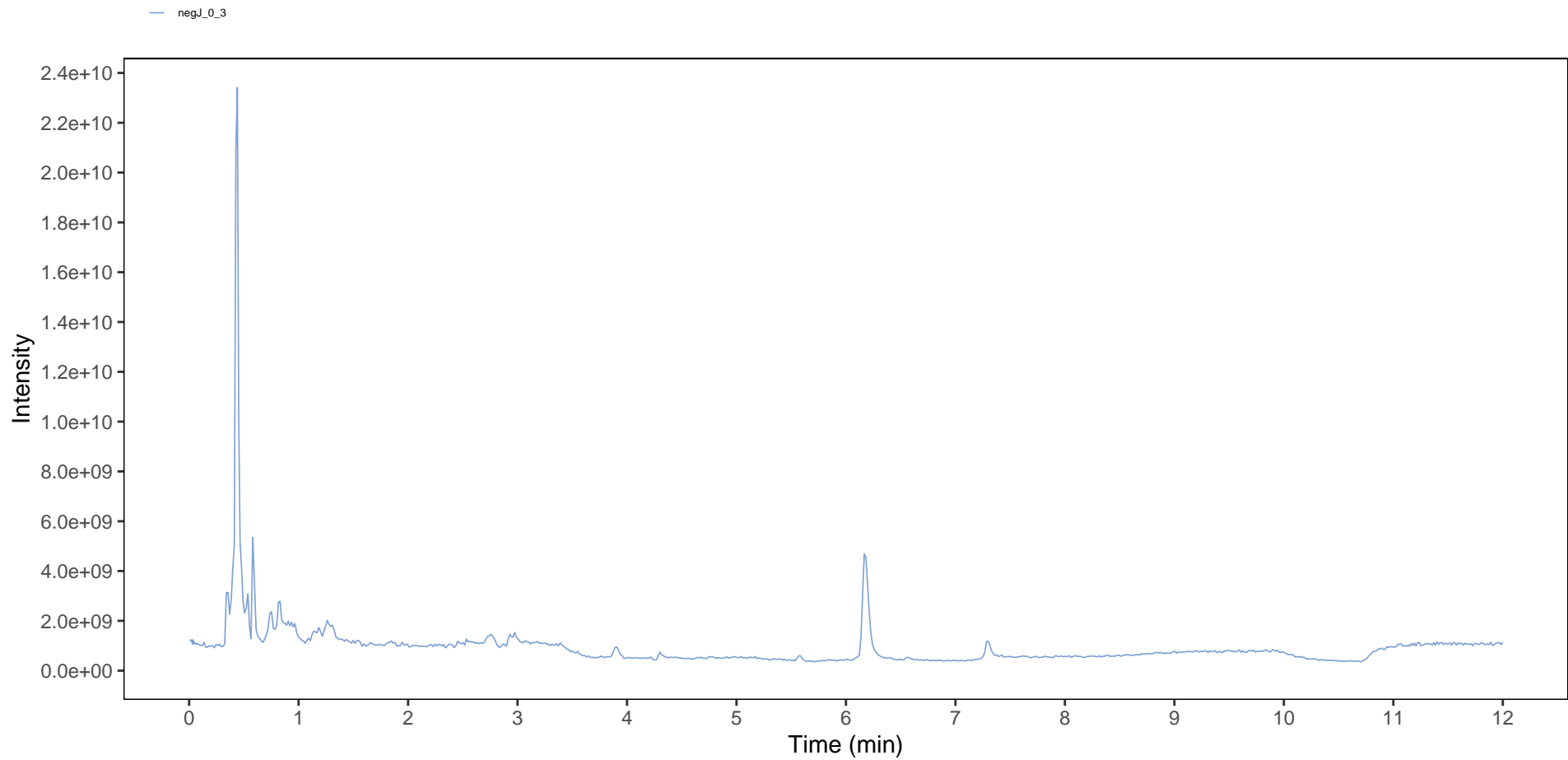

negJ\_3\_1

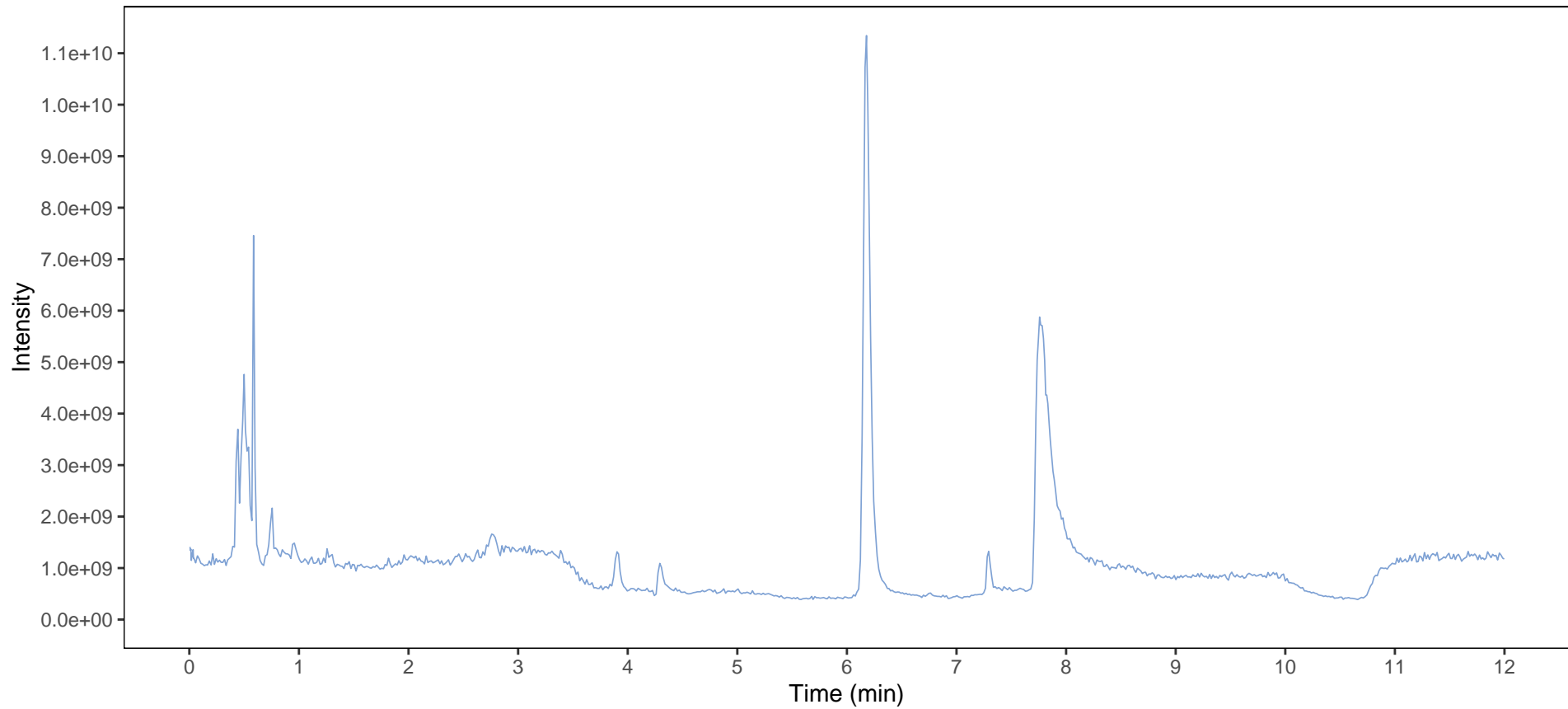

negJ\_3\_2

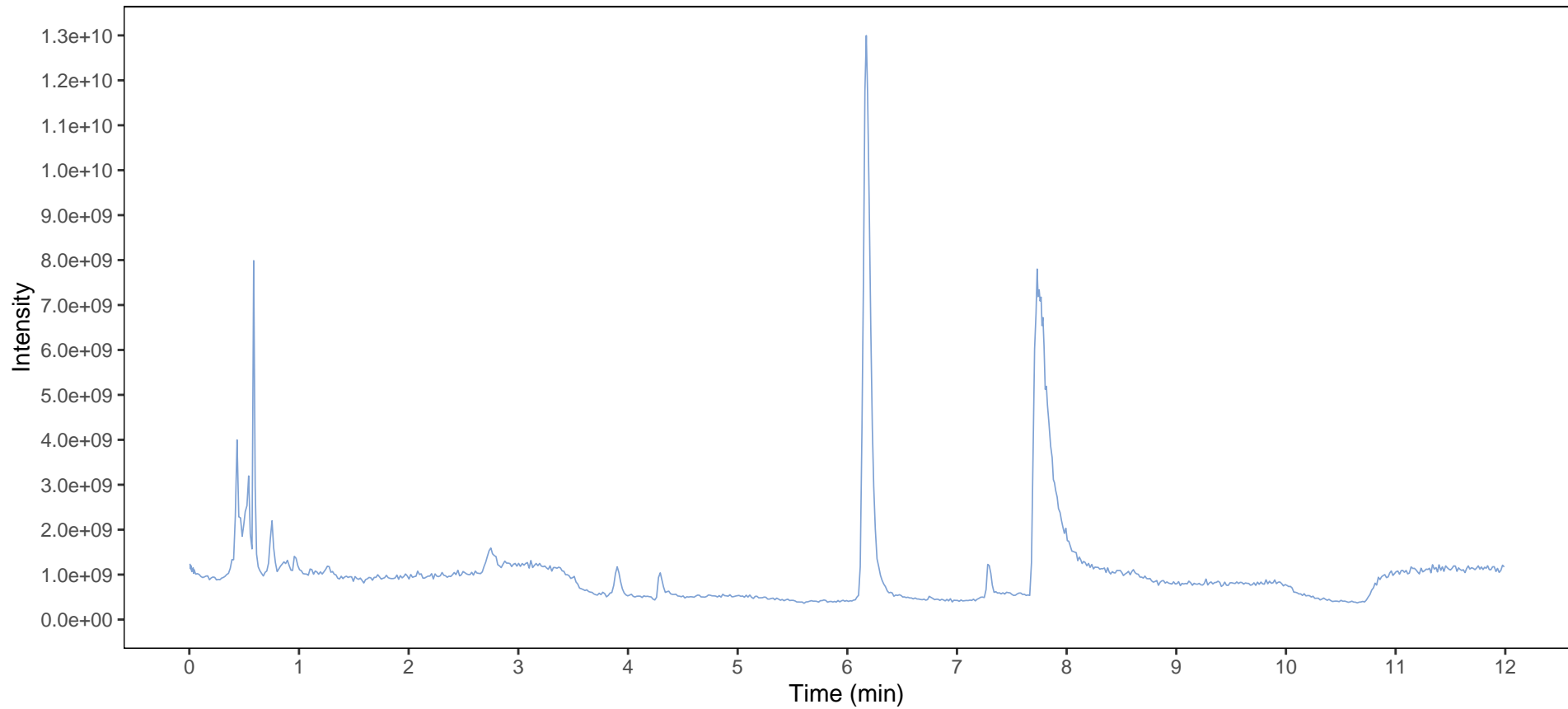

negJ\_3\_3

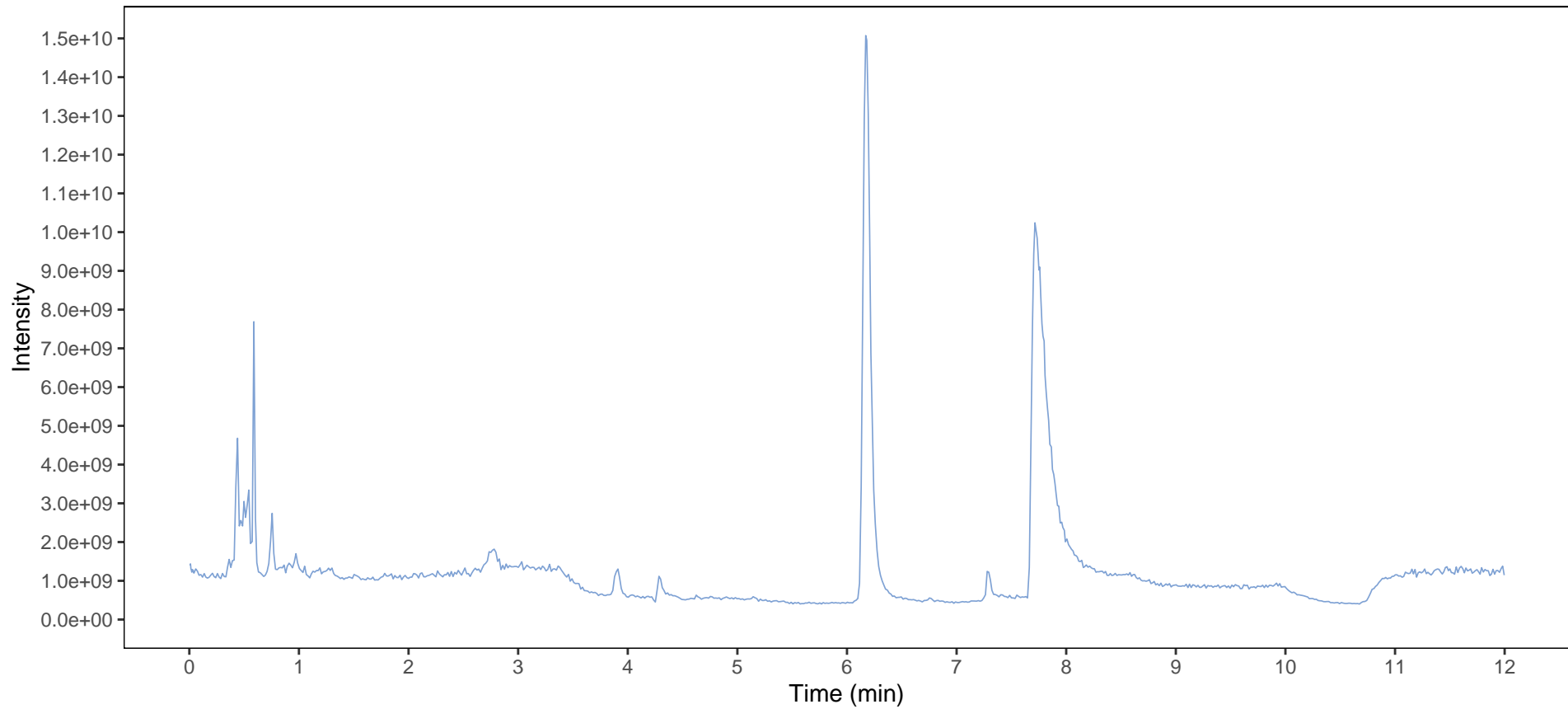

negJ\_24\_1

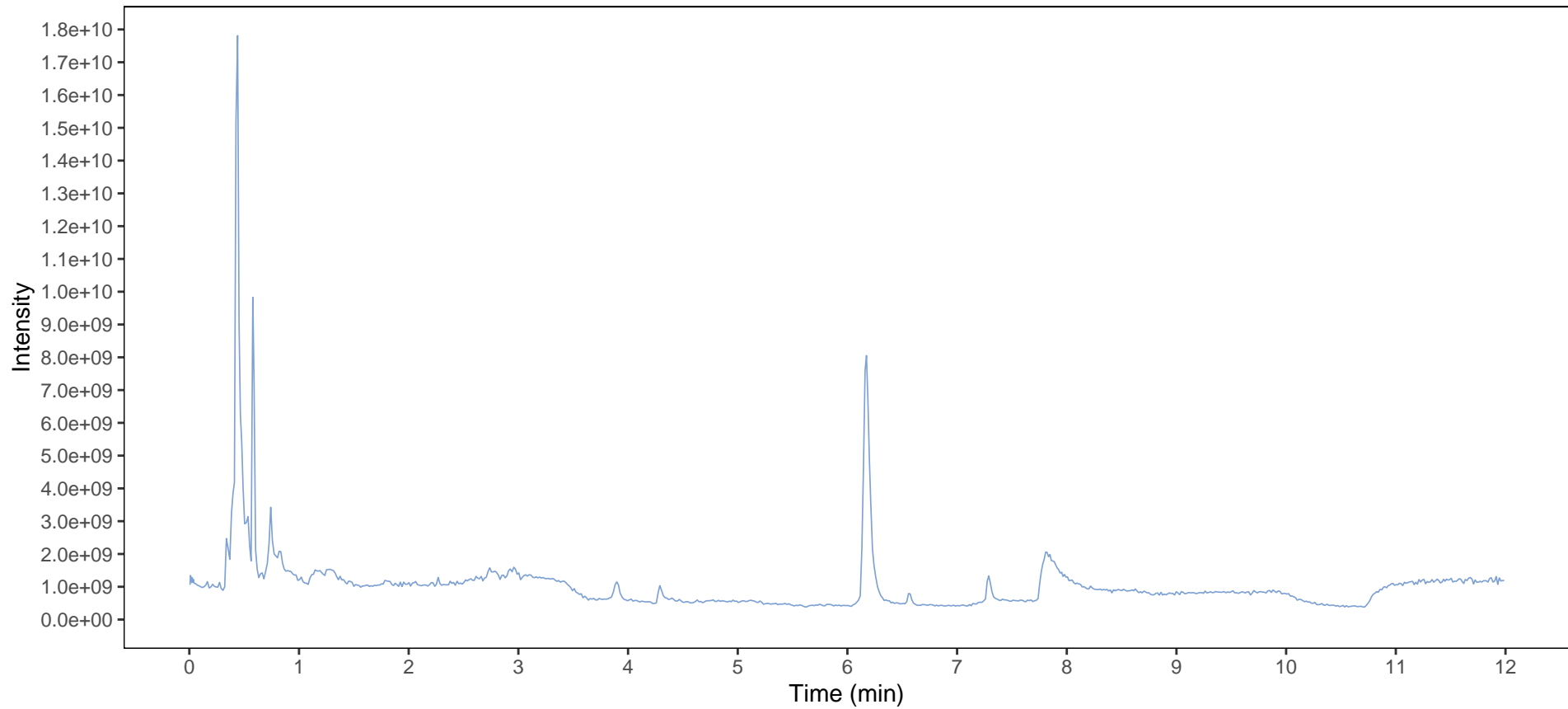

negJ\_24\_3

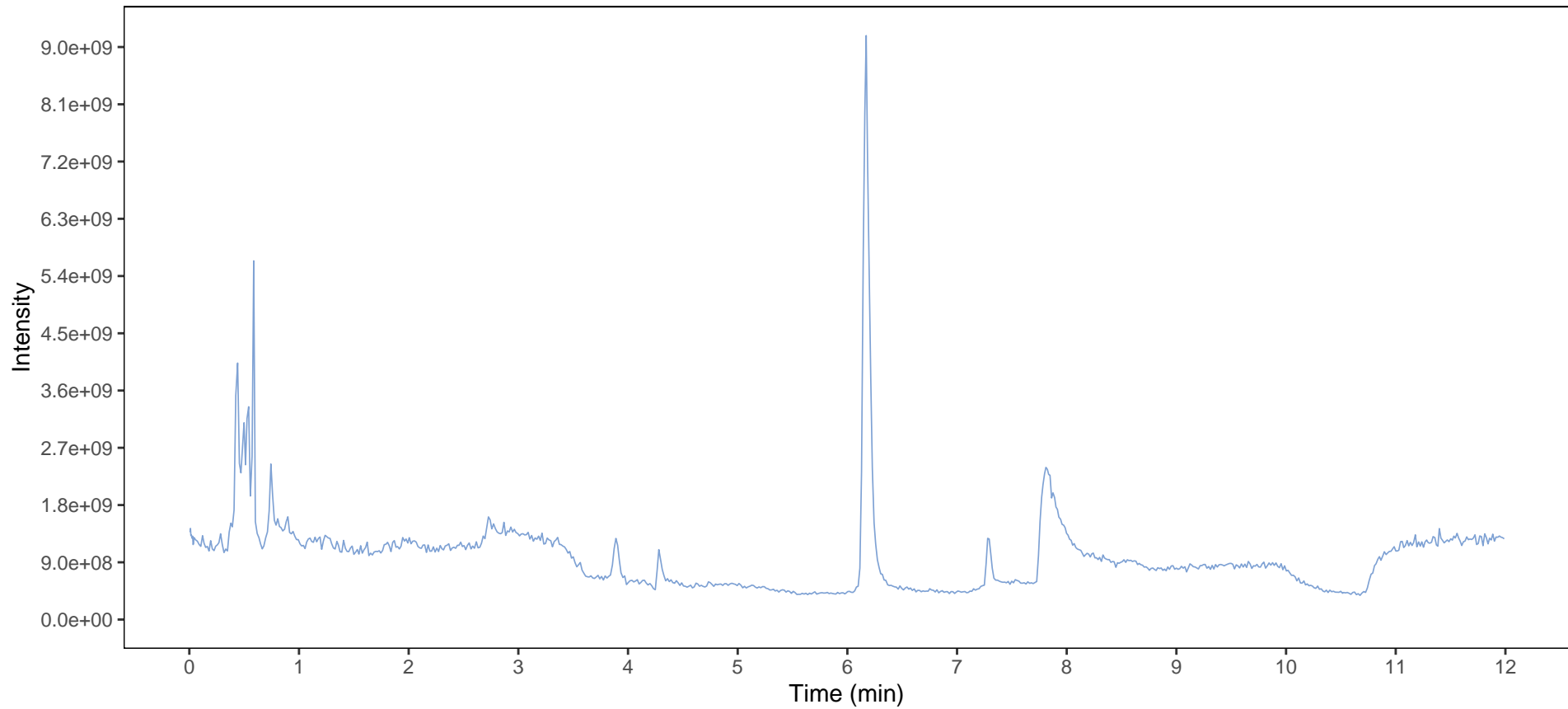

negJ\_24-2

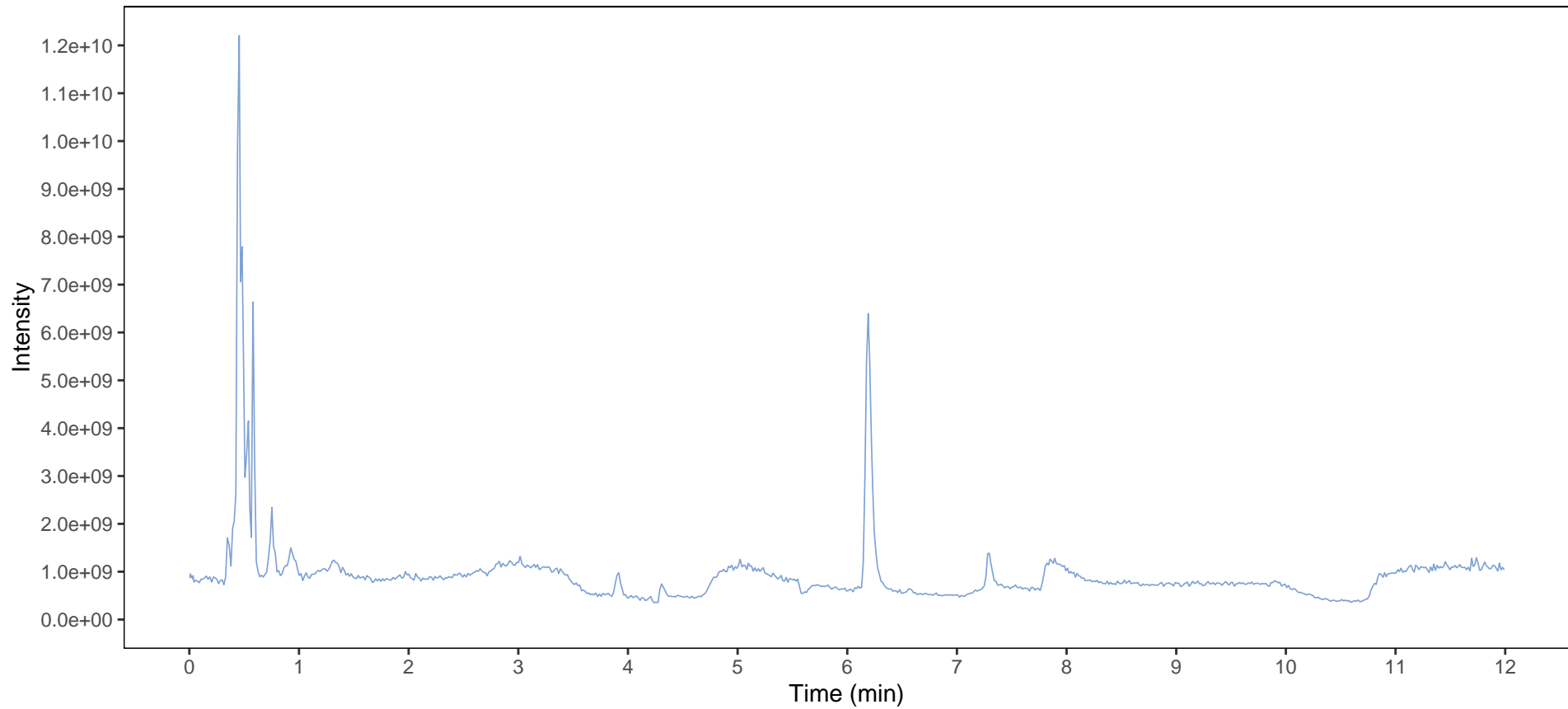

Supplement: SUPPLEMENTARY FIGURE S5 — Volcano plot of differential expressed metabolites induced by (A) desiccation treatment and (B) SMP storage. [file Data_Sheet_2.PDF]

posJ\_0\_1

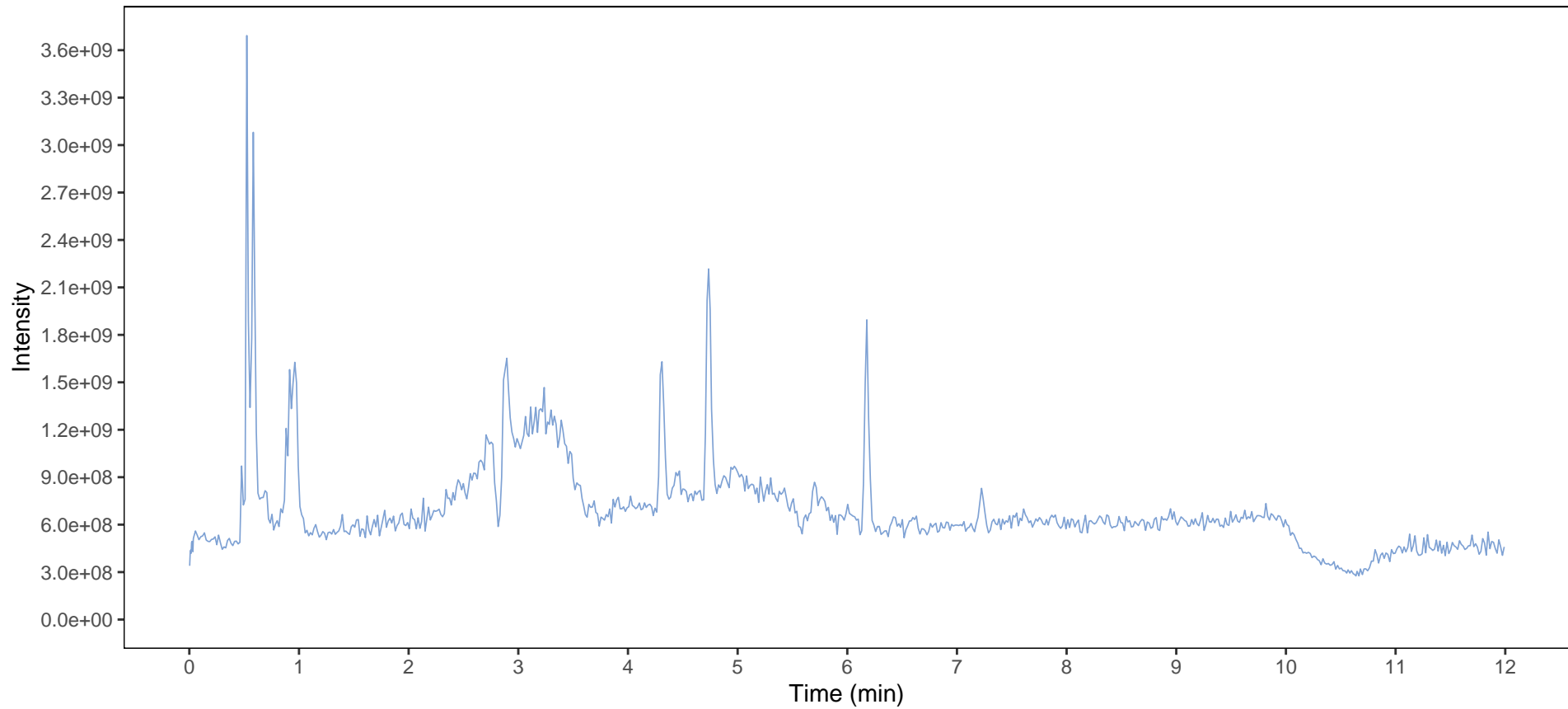

posJ\_0\_2

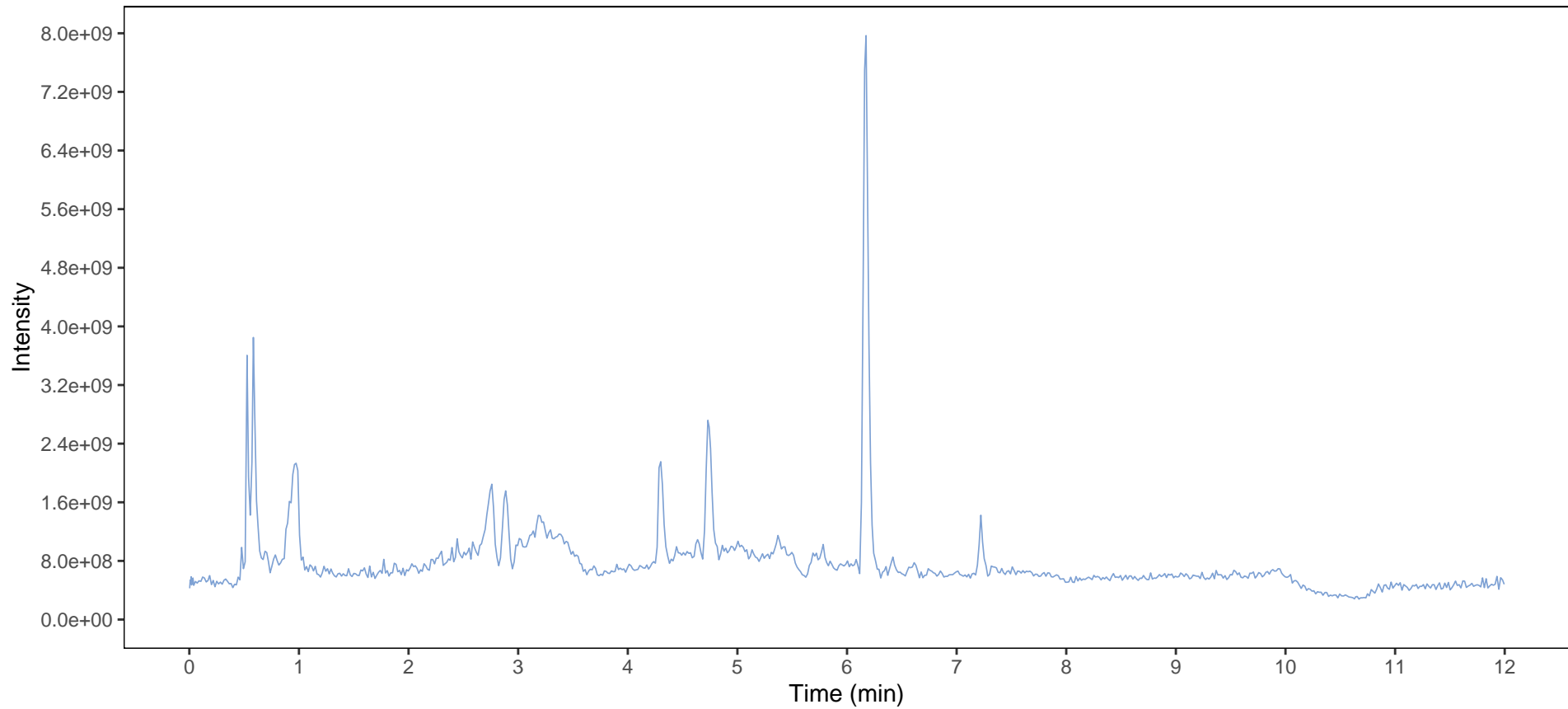

posJ\_0\_3

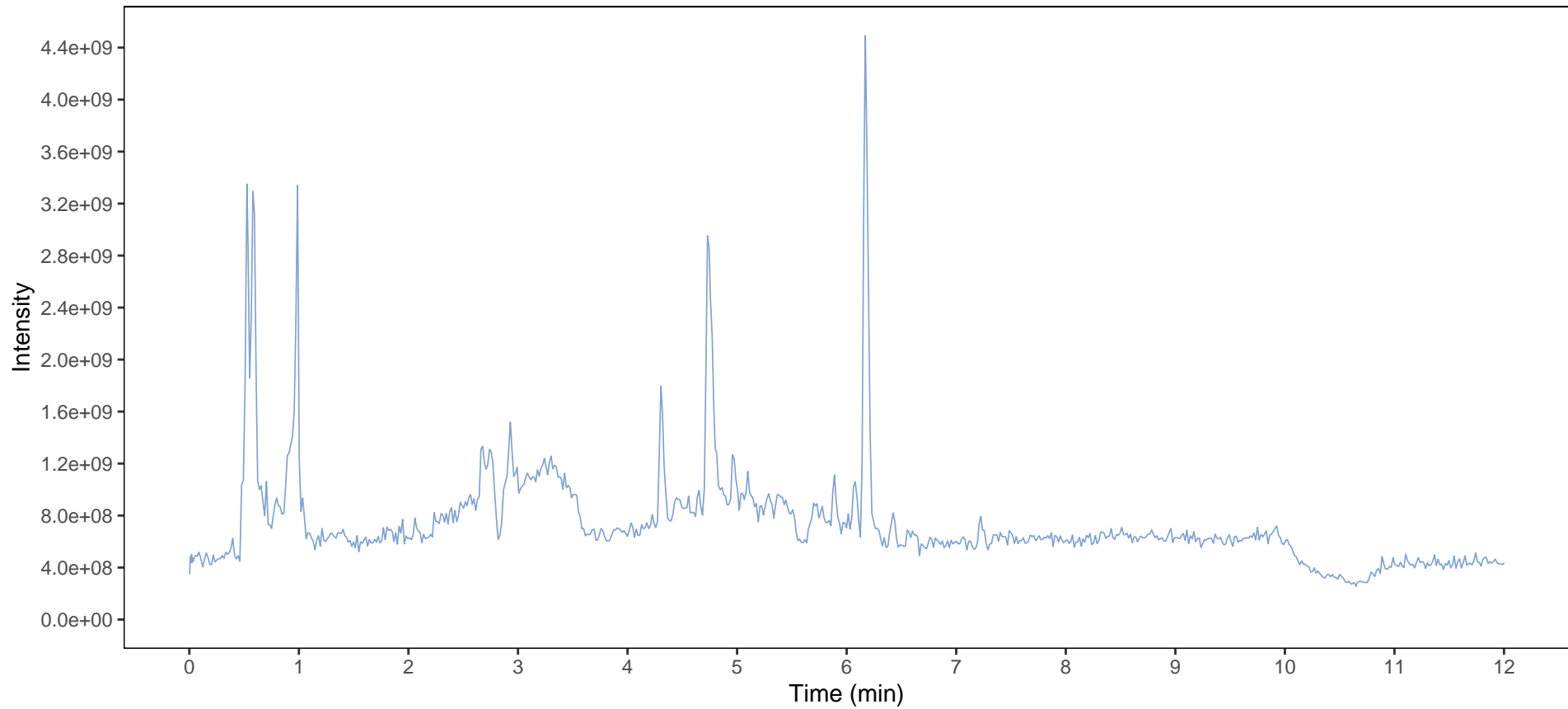

posJ\_3\_1

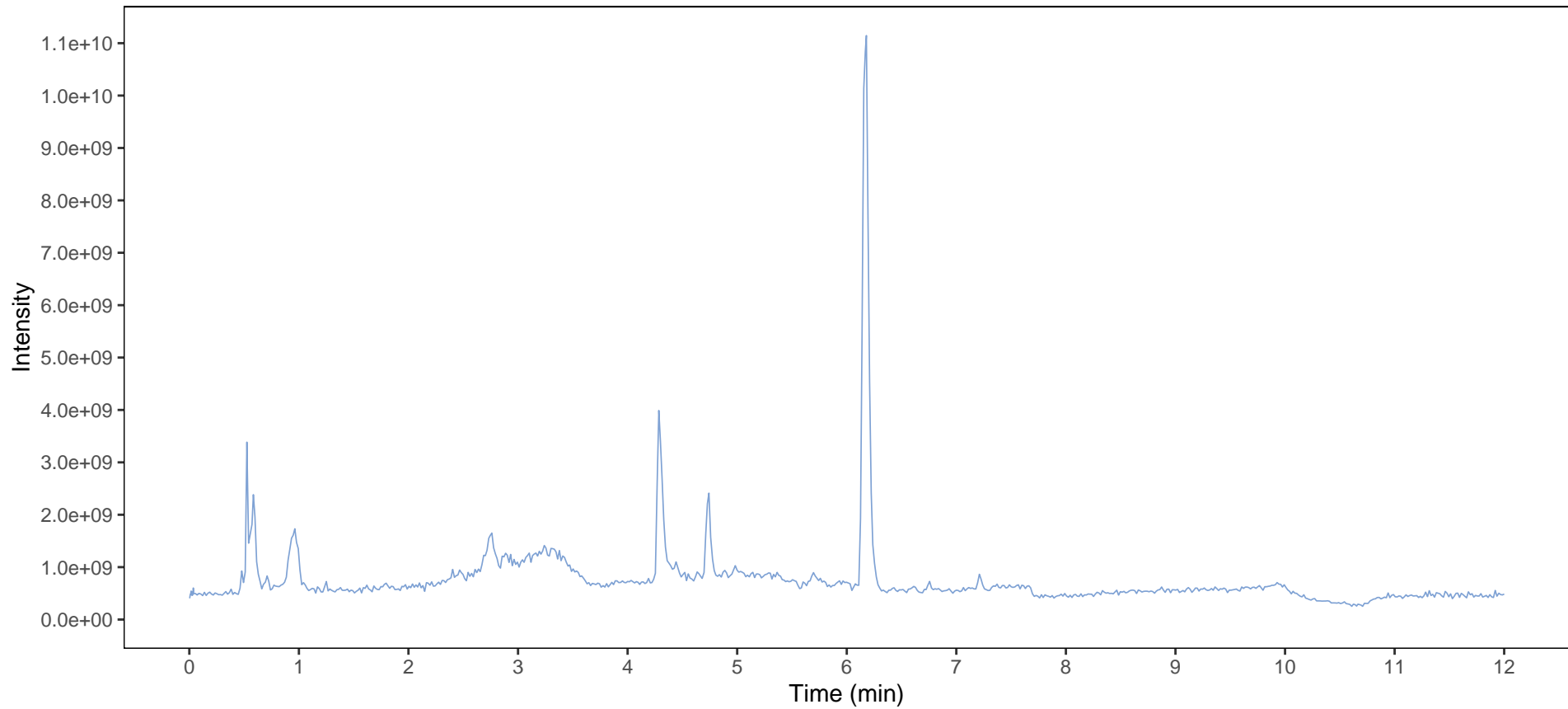

posJ\_3\_2

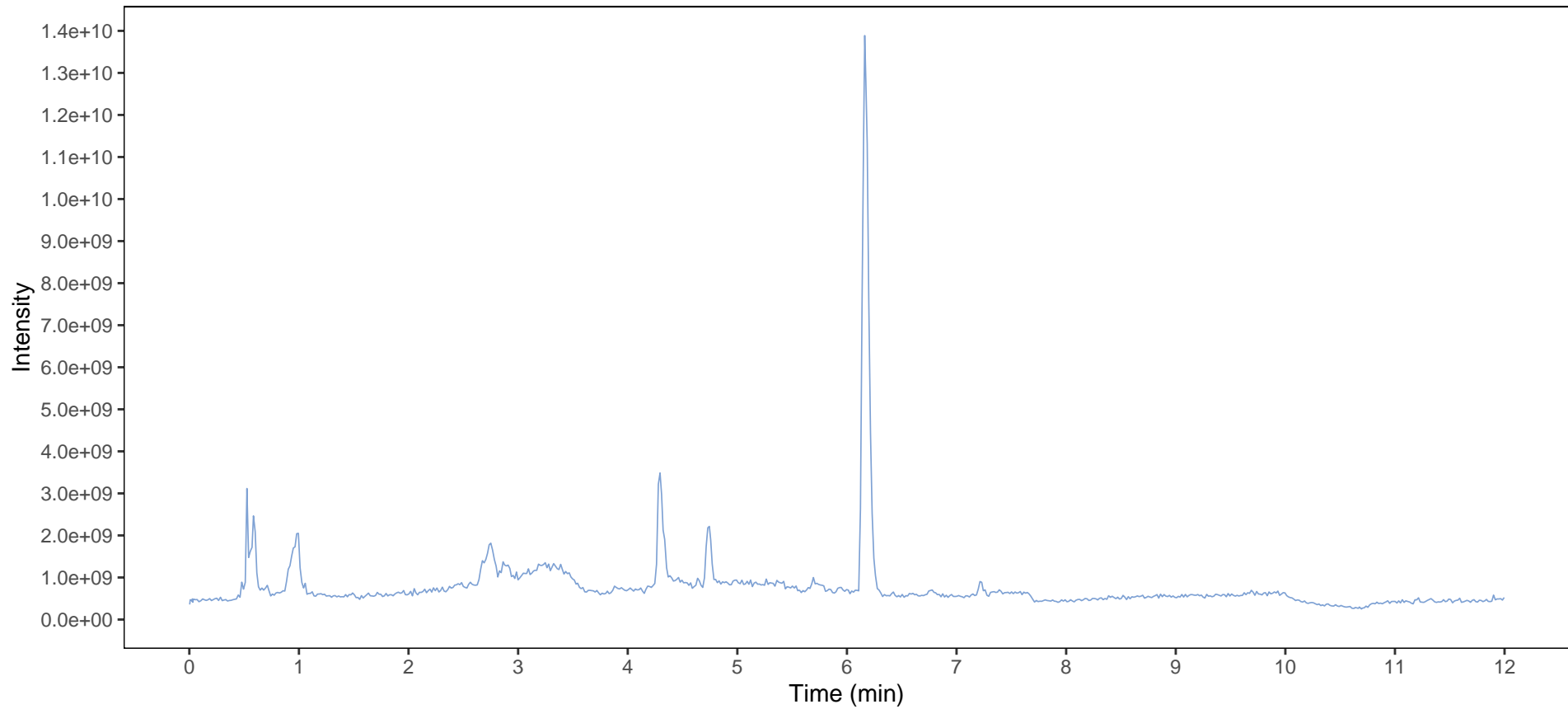

posJ\_3\_3

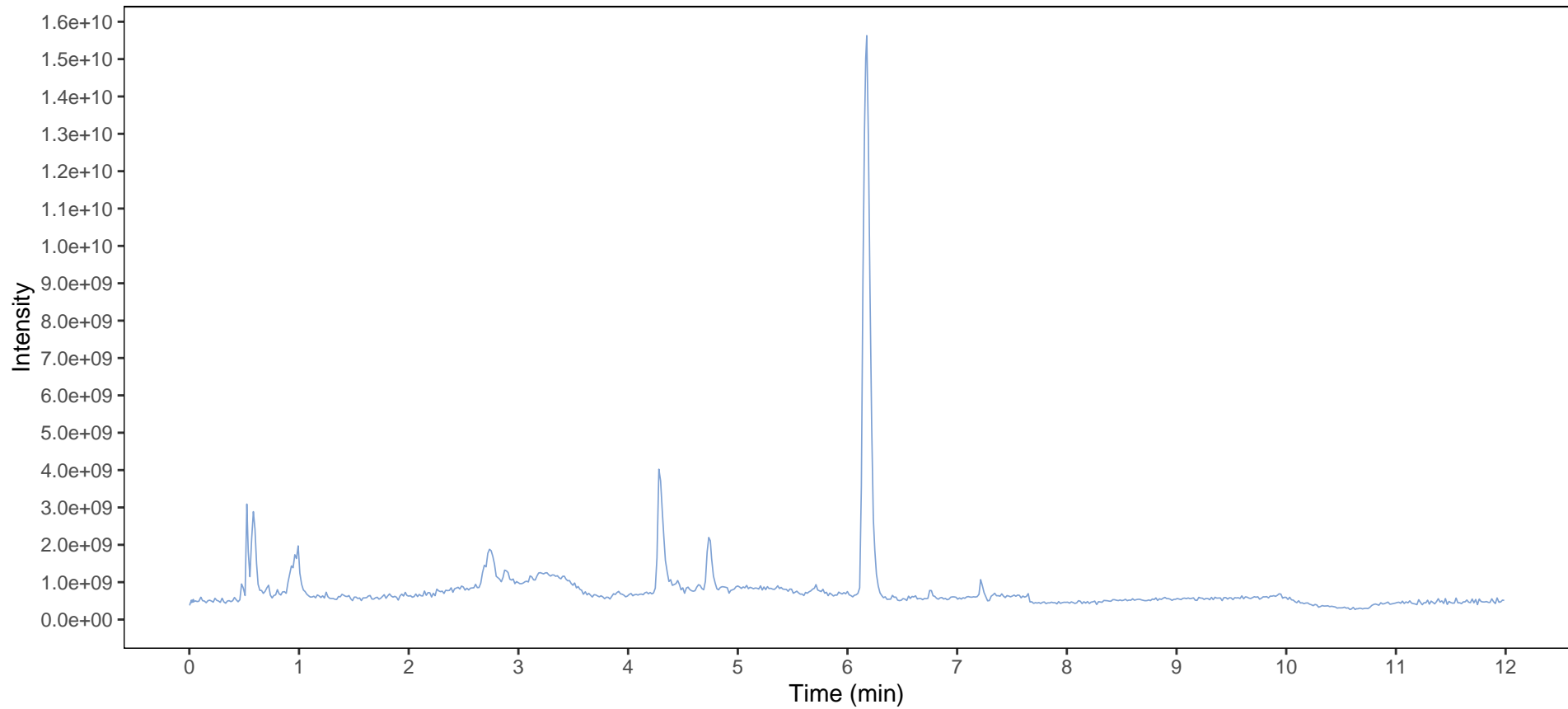

posJ\_24\_1

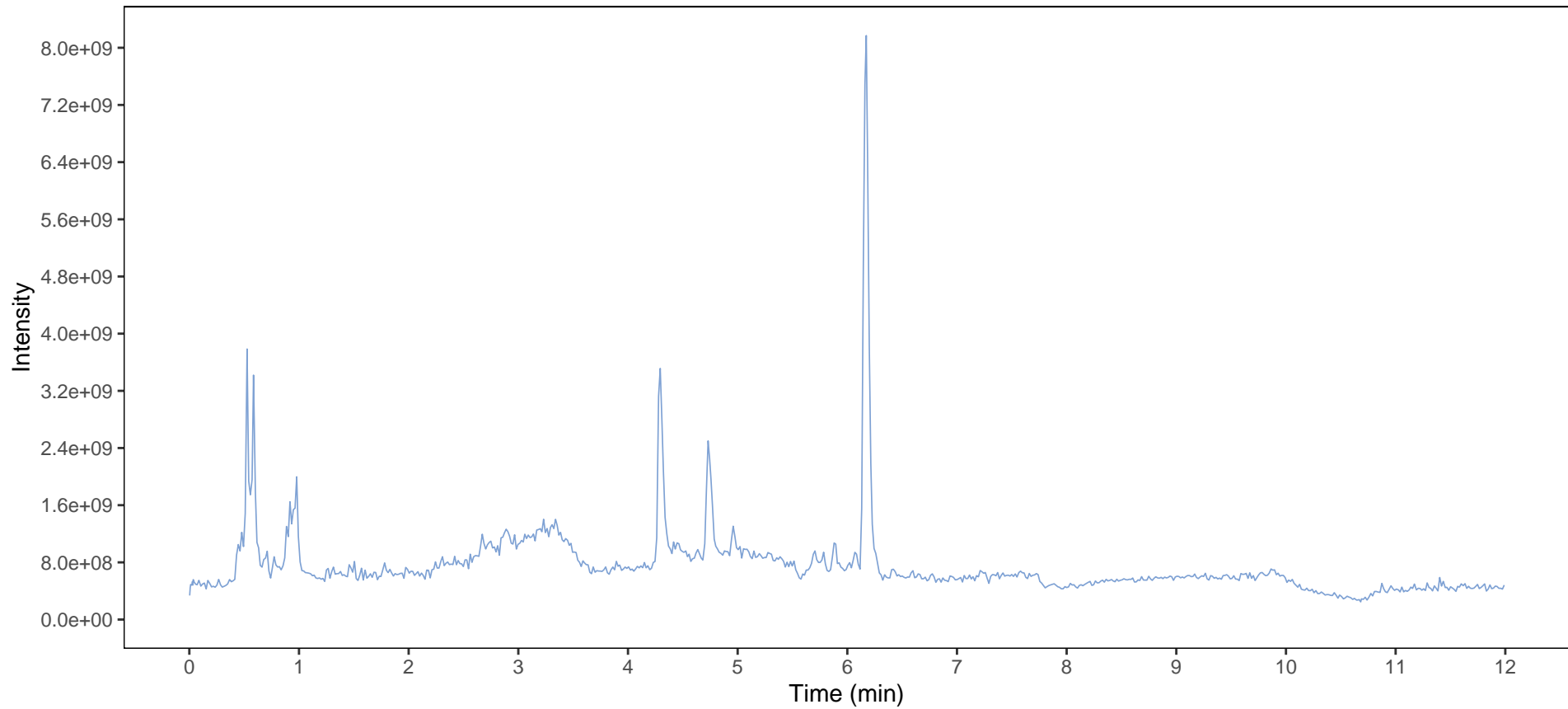

posJ\_24\_3

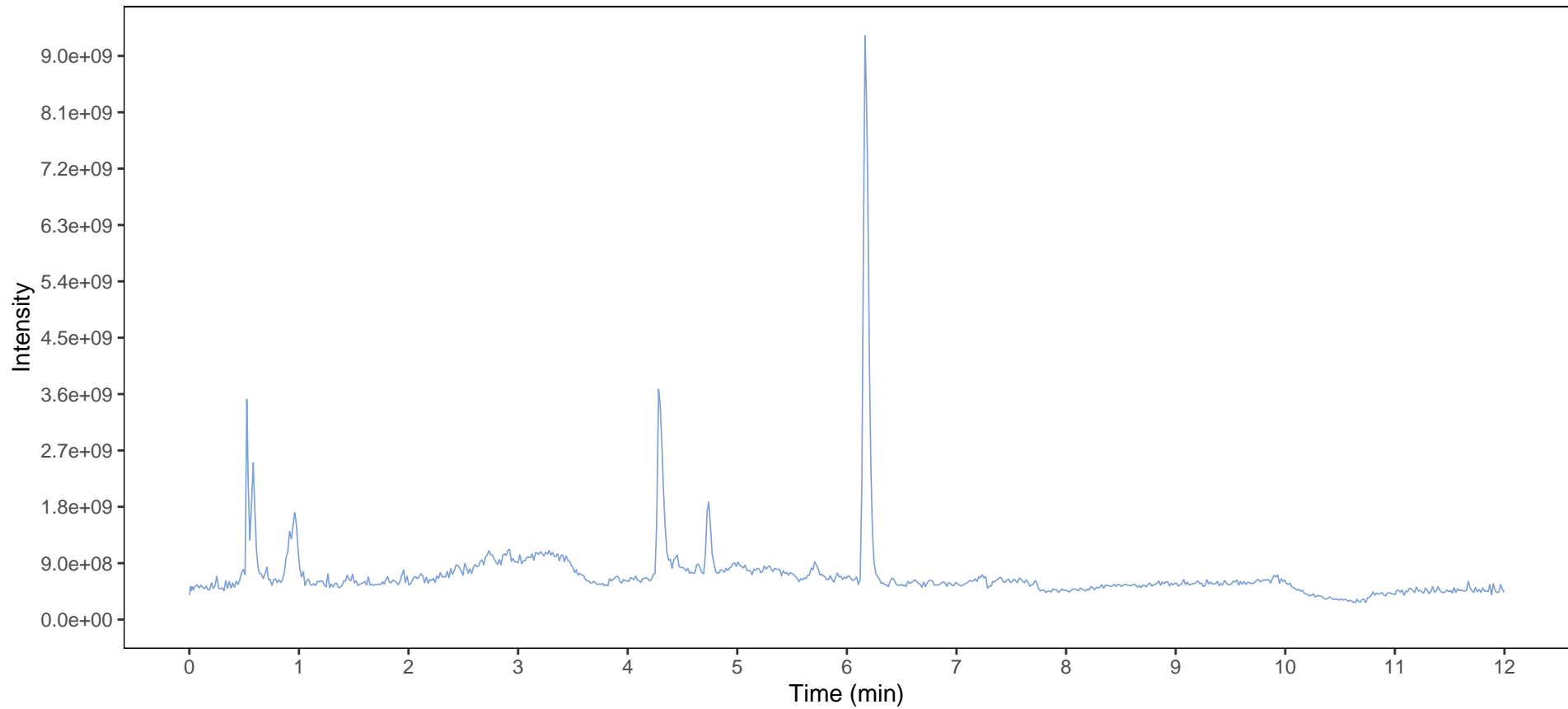

posJ\_24-2

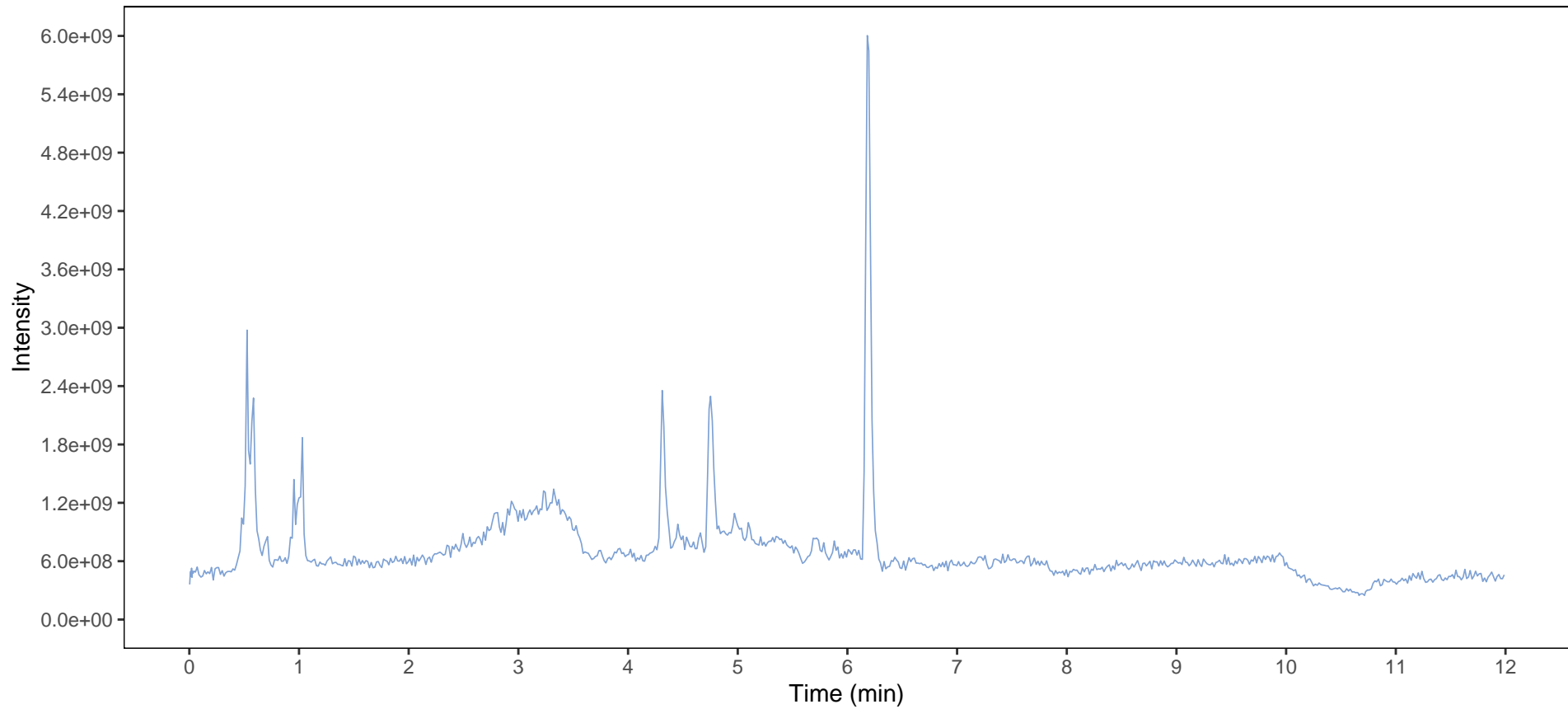

Supplement: SUPPLEMENTARY FIGURE S6 — Heatmap of hierarchical clustering analysis of all the identified differentially expressed metabolites in the 0-, 24h and 3-month samples. [file Data_Sheet_3.PDF]
